# HLA DP/DRA molecule regulates systemic inflammation and neuroinflammation, aggravates cognitive impairment and long-term anxiety in murine model of sepsis-associated encephalopathy

**DOI:** 10.3389/fimmu.2026.1798003

**Published:** 2026-06-16

**Authors:** Feng Li, Bowen Niu, Yu Huang, Mengmin Zhu, Lingling Liu, Lixiang Chen, Hua Yang, Boyin Qin, Xiaohui Zhou

**Affiliations:** 1 Department of Laboratory Animal Science, Shanghai Public Health Clinical Center, Fudan University, Shanghai, China; 2 Department of Biology, College of Life Sciences, Shanghai Normal University, Shanghai, China

**Keywords:** anxiety, cognitive impairment, hippocampus, HLA-DP401, HLA-DRA, inflammation, MHC class II, sepsis

## Abstract

Sepsis-associated encephalopathy (SAE) is a severe and common neurological complication of sepsis, characterized by symptoms ranging from mild confusion, delirium, deep coma, and severe cognitive dysfunction. Previous epidemiological and bioinformatics studies have revealed that HLA DP and DRA molecule play a pivotal role during sepsis. However, the mechanism by which these class II molecule contribute to cognitive impairment in SAE remains unclear. using the peritoneal contamination and infection model (PCI) model in humanized transgenic HLA-DP401/DRA-IAβ^-/-^ genotypes mice, we aimed to investigate the effects of HLA class II haplotypes/alleles on sepsis and elucidate the underlying mechanism leading to cognitive impairment. Our results indicated that the introduction of HLA DP/DRA molecule significantly increased mortality, exacerbated clinical symptoms, and elevated inflammatory cytokine responses in both serum and hippocampal tissue of septic mice. Cecal slurry (CS) injection induced robust microglia activation and severe pathological damage of hippocampus. Furthermore, transcriptome analysis revealed numerous differentially expressed genes (DEGs) and prominent mitochondrial dysfunction in HLA-DP/DRA-IAβ^-/-^ mice subjected to PCI. Notably, CS injection up-regulated AMPK-α phosphorylation in IAβ^-/-^ mice but not in HLA DP/DRA-IAβ^-/-^ mice. Consistently, sepsis induced persistent neurocognitive deficits and long-term anxiety-like behaviors in HLA DP/DRA-IAβ^-/-^ PCI mice. In conclusion, these data provide direct evidence that HLA class II molecules modulate the host response to sepsis and highlight a critical role of HLA-DP/DRA in exacerbating the severity of systemic infection. The introduction of the HLA-DP and HLA-DRA genes synergistically upregulated systemic and hippocampal inflammatory cytokines, worsened clinical outcomes, impaired memory performance, and exacerbated long-term anxiety-like behaviors.

## Introduction

Sepsis is a life-threatening organic dysfunction caused by an overwhelming and dysregulated host response to inflammation (1) Epidemiological studies have revealed an incidence rate of 437 hospital-treated sepsis patients per 100,000 person-years, with an estimated global incidence of 31.5 million sepsis cases and 5.3 million deaths annually (2–5). Up to 70% of sepsis patients develop sepsis-associated encephalopathy (SAE), which is defined as diffuse cerebral dysfunction, frequently develops long-term cognitive impairments, including deficits in attention, memory, executive function, and information processing speed (4–7).

The pathophysiology of SAE is multifactorial, involving severe systemic inflammation, blood-brain barrier disruption, the release of cytokines, chemokines, oxygen radicals, and reactive nitrogen intermediates, microglial activation, ischemic/hemorrhagic lesions, and altered cerebral microcirculation (8–11). Additionally, infiltration of peripheral immune cells into the central nervous system (CNS) also contributes to SAE pathogenesis (12). However, the neuronal pathophysiological mechanisms underlying SAE and post-sepsis cognitive decline remain incompletely understood.

The diversity of major histocompatibility complex (MHC) molecular polymorphisms is strongly linked to a wide range of human diseases, including autoimmune and infectious diseases (13–15). Published data prompt that the HLA class II molecule may act as key regulators of sepsis-induced neuroinflammation. For example, the HLA-DR expression levels provide critical insights into predicting mortality and the risk of secondary infections in sepsis patients (16). HLA-DP molecules are constitutively expressed on antigen-presenting cells and B cells, and their expression is upregulated under inflammatory conditions (17–20). Specific HLA-DP allotypes, along with the expression levels of HLA-DP molecules, have been associated to many human diseases, including graft-versus-host disease (21), hepatitis B virus (HBV) infection (22, 23), HIV (24, 25) and autoimmune diseases (15, 18, 26). Additionally, interactions between the NK cell receptor NKp44 and HLA-DP401 can trigger functional NK cell responses, implicating HLA class II molecules in the regulation of the innate immune response (27). HLA-DP401 molecules expressed on intestinal epithelial cells can activate NKp44^+^ NK cells by binding to NKp44; activated NK cells exhibit increased degranulation and tumor necrosis factor (TNF) production, leading to intestinal epithelial damage (28). Effector CD8^+^ T cells express HLA-DP molecules; HLA-DP^+^ CD8^+^ T cells can activate NKp44^+^ NK cells through an HLA-DP haplotype-dependent manner, which in turn regulates CD8^+^ T cell expansion and clonality (29). Several studies have revealed that HLA-DPB1 correlates more strongly with the adaptive immune response in sepsis (30–36). Previous studies have also shown the upregulation of MHC II molecule expression in the central nervous system (CNS) under pathological conditions (37–39). Class II transactivator (CIITA) in the CNS drives neuroinflammation and neurodegeneration in an alpha-synuclein model of Parkinson’s disease (40).

To provide an *in vivo* validation of these findings, an appropriate animal model of sepsis is needed to investigate the role of HLA-DP and -DRA molecules. The HLA class II transgenic mice have contributed significantly to our understanding of the mechanisms involved in Gram-positive bacterial toxic shock. Several studies have provided evidence for a direct role of HLA class II molecules in modulating host responses to superantigens (SAgs) and highlighted the allelic variation in HLA class II molecules play a dominant role in exacerbating the severity of systemic infections caused by Gram-positive bacteria (41–45). Humanized HLA-A11/DR1 mice exhibit a prolonged infection accompanied by impaired resolution of infection and increased bacterial load following Streptococcus suis (S. suis) infection (46).

In this study, we employed an HLA-DP/DRA transgenic (Tg) mouse model (based on the HLA-DP401/DRA0101 genotypes) (45, 47), an allele combination present in 20-60% of the global population (48, 49). We comprehensively investigated the role of human HLA II molecules in sepsis and evaluated differences in inflammatory responses, hippocampus pathology and behavioral alternations between HLA II transgenic mice and murine H2-deficient mice. The work presented here demonstrates that HLA DP/DRA molecules directly contribute to sepsis severity and outcome and supports previous epidemiological and bioinformatics findings linking the HLA-DP/DRA allele to elevated sepsis susceptibility.

## Materials and methods

### Mice and ethics statement

HLA-transgenic (HLA-tg) mice deficient in MHC class II (IAβ^−/−^) and expressing HLA-DP401/DRA01, HLA-DP401 and HLA-DRA01 alleles were generated in our laboratory, as previously published (45, 47). In brief, double-transgenic HLA-DP401/DRA01 mice were generated by crossing HLA-DP401-IAβ^−/−^ mice with HLA-DRA01-IAβ^−/−^ mice. HLA-DP401/DRA01-IAβ^−/−^, HLA-DP401-IAβ^−/−^, HLA-DRA-IAβ^−/−^ and IAβ^−/−^ mice were bred in the animal facility of Shanghai Public Health Clinical Center (SHPHC). In the following experiments, all transgenic mice were randomly allocated to groups with an equal male/female ration (1:1). Mice weighed 20–25 g and were 8–12 weeks old. Each group contained 15–20 mice per batch. C57BL/6J (wild-type [WT]) mice were purchased from Shanghai SLAC Laboratory Animal Co., Ltd. All infection experiments were carried out in the Biosafety Level 2 Laboratory of SHPHC (Approval No. 2025-A016-01).

### Peritoneal contamination and infection model

The PCI model was adapted from a previous report (50). In brief, the cecal slurry (CS) were collected from 8-10-week-old female C57BL/6J mice. To minimize individual variation, cecal contents from multiple donors (n ≥ 10 per batch) were pooled, homogenized, weighed, diluted to 100 mg/mL with 15% (v/v) sterile glycerol (in PBS). To generate the PCI model in recipient mice, the animals received an intraperitoneal injection at 1.2 mg/kg. Six hours post-injection, meropenem (20 mg/kg, MB1129, Meilunbio) was administered intraperitoneally. Thereafter, these mice received a inject every 12 hours for 7 consecutive days. No additional analgesics or fluid resuscitation were administered beyond standard antibiotic support (meropenem), consistent with widely accepted protocols for the PCI model in sepsis-associated encephalopathy research (50, 51). Murine sepsis score (MSS) were monitored daily for 7 days, according to previously reported methods (52). Animals with scores >14 points during the first 3 days post-infection were defined as severe sepsis cases and subsequently subjected to SAE analysis.

### 16S rDNA amplicon sequencing

16S rDNA sequencing was performed by Beijing Novogene Biotechnology Co., Ltd. Briefly, bacterial DNA was extracted from cecal slurry (CS) according to the manufacturer’s protocol. The 16S V4 region DNA was then amplified using specific primer (515F: GTGCCAGCMGCCGCGGTAA; 806R: GGACTACHVGGGTWTCTAAT), followed by purification with magnetic beads and quantification via qPCR to construct the 16S rDNA library. The library was sequenced on the Illumina MiSeq platform (CA, USA). The raw data underwent demultiplexing, paired-end read merging, and quality control to generate clean data. To investigate the species composition of each sample, effective sequences from all samples were clustered into Operational Taxonomic Units (OTUs) at 97% identity. Representative sequences of OTUs were then taxonomically annotated using the Silva 138.1 database (http://www.arb-silva.de/). Based on the OTU annotation results and sample abundance profiles, species abundance tables at the kingdom, phylum, class, order, family, genus, and species levels were obtained.

### RNA isolation and quantitative real-time PCR

TRIzol reagent (19211ES60), cDNA Reverse Transcription Kit for qPCR (Cat.No. 11123ES60) and SYBR Green qPCR Master Mix (Cat.No. 11201ES08) were purchased from Yeasen Biotechnology (Shanghai, China) Co., Ltd. Total RNA of HMC3 cells or hippocampus tissues were isolated using TRIzol reagent. The mRNA levels were assessed using RT Master Mix and SYBR Green qPCR Master Mix based on the manufacturer’s recommended protocol. Gene expression was measured using a fluorescence quantitative PCR system (Bioer, Hangzhou, LineGene 9600). The relative quantification of gene expression was determined using the ΔΔCT method, with β-actin serving as the internal reference control. Primer sequences of targeted genes are listed in **Supplementary Table 1**.

### Enzyme linked immunosorbent assay

Serum cytokine levels were measured according to previously established methods (45). Briefly, 24 hours post CS infection, blood was collected from the retro-orbital plexus and left to coagulate at RT for 30 min, followed by centrifugation at 1000 ×g for 10 min to obtain serum. Tumor necrosis factor-α (TNF-α; SEKM-0034), interleukin-1β (IL-1β; SEKM-0002) and IL-6 (SEKM-0007) concentrations were determined with commercially available ELISA kits (Solarbio, Beijing, China) according to the manufacturer’s instructions. Optical density was measured at 450 nm using a Luminex 100 platform (Bio-Rad, Hercules, CA, USA).

### Western blot

Proteins extraction of hippocampal tissues were performed in a kit supplemented with a protease inhibitor cocktail according to the manufacturer’s instructions (Cat# PC201Plus, Epizyme Biotech, Shanghai, China). Solubilized proteins were separated by SDS-PAGE, transferred onto nitrocellulose membrane (ISEQ00010, Merck MilliPore, UK), and immunoblotted as previously described (45). The membranes were then blocked and incubated with primary antibodies overnight at 4 °C (**Supplementary Table 2**). After washed and incubated with the corresponding HRP-conjugated secondary antibodies (HS101-01, TransGen, China) for 2 h, immunoreactive bands were detected with an enhanced chemiluminescence detection reagent (GE Healthcare, Piscataway, NJ, USA) and images captured with G:BOX GENI (Syngene, Synoptics Ltd, Cambridge, England) according to the manufacturer’s instructions. Band intensities were quantified by spot densitometric analysis using image J software, and results were normalized to actin levels and reported as relative intensities to controls.

### Immunofluorescence

The brains were fixed in 4% PFA for 24 h and immersed in 30% sucrose solution for 24 h. The embedding brains in OCT were cut into 20μm sagittal sections on a cryostat (CM1950, Leica). The sections were washed with PBS three times and then were blocked with 5% bovine serum albumin (BSA) for 5 h at 4 °C, as previously described (53). Subsequently, the sections were incubated with primary antibodies overnight at 4 °C (**Supplementary Table 2**). After three PBS washes, the sections were incubated with Alexa Fluor 488-conjugated goat anti-rabbit IgG (GB25303, Servicebio) for 1 h at RT. Nuclei were stained with Hoechst 33342 (1:1000, 40732ES03, Yeasen) for 30 min at RT. After mounted with anti-fade mounting medium (P0126, Beyotime), images were acquired using a Leica SP8 confocal microscope with z-stack scanning at 1-μm intervals, and the final images were generated by maximum intensity projection (MIP).

### Nissl staining

After fixation in 4% PFA, the brains were cut evenly into two part along the midline, dehydrated, cleared, and embedded in paraffin. Paraffin-embedded tissues were cut into 5-μm sections by a microtome (RM2016, Leica). The slices were dewaxed, rehydrated, and stained with toluidine blue (G3668, Solarbio) for 3 min. After rinsing, differentiated, dried, the sections were immersed in dimethyl benzene for 3 min twice, sealed with neutral balsam. Images were acquired using a NIKON DS-U3 microscopy system.

### Golgi staining

Brains were immersed in Golgi fixative solution (G1069, Servicebio) for 24 hour at RT, transferred to Golgi staining solution (G1069-1, Servicebio) and stored at RT in the dark for 14 days. Subsequently, the samples were moved to tissue processing solution (G1069-3, Servicebio) for 3 days at 4 °C. Coronal sections (60 μm thick) were prepared using a vibrating microtome (VT1000S, Leica), stained in Golgi staining developer solution (G1069-2, Servicebio) for 30 min and then coverslipped with glycerol.

Whole-slide imaging was performed using a 3DHISTECH digital slide scanner, and analyzed using CaseViewer software. Neurons with clearly visible black-stained somata were selected for further evaluation. Axons exhibiting abrupt termination or discontinuous segments with missing intermediate regions were classified as axonal fragmentation. For each mouse, 30 randomly selected dendrites were analyzed, and dendritic spine density was quantified using ImageJ.

### Transmission electron microscopy

Mice were anesthetized with avertin (500 mg/kg), hippocampus were rapidly extracted and cut into 1-mm³ pieces and fixed in electron microscopy fixative (P1126, Solarbio) at 4 °C for 2 hours. Post-fixation was performed with 1% osmium tetroxide at RT for 2 hours, followed by graded ethanol dehydration and embedded in acetone-resin mixture. Ultrathin sections (60 nm) were cut using an ultramicrotome (UC7, Leica), double-stained with uranyl acetate and lead citrate, and imaged using a transmission electron microscope (HT7700, Hitachi).

### HMC3 cells culture

Human microglial clone 3 cell line (HMC3) cells were obtained from the American Type Culture Collection (ATCC). After thawing, cells were passaged 2–3 times and stored frozen at -80 °C. Then, frozen cells were thawed and subcultured 1–2 times before use in stimulation assays. HMC3 cells were cultured in Dul-becco modified Eagle’s medium (DMEM, Gibco, Carlsbad, CA, USA) supplemented with 10% fetal bovine serum (FBS, Sijiqing, Hangzhou, China), 50 U/ml penicillin, and 50 μg/ml streptomycin at 37 °C in a humidified atmosphere with 5% CO2. HMC3 Cells were seeded at 1×10^6^ cell density in 6-well plates. After 24 h, HMC3 cells were treated with 200 ng/mL LPS (L4130, Sigma-Aldrich, USA) and 20 ng/mL IFN-γ (91207ES20, Yeasen, China) for 24 h. Later, cells and supernatant were collected for further measurements. Primer sequences are listed in **Supplementary Table 1**.

### Elevated plus maze

The elevated plus maze consisted of two opposing open arms (31 × 5.8 cm) and two enclosed arms (31 × 5.8 cm) extending from a central platform (5.8 × 5.8 cm), elevated 50 cm above the floor (53). Each mouse was placed on the central platform facing an open arm, and its behavior was recorded for 5 minutes and analyzed using SA211 (Sansbio, China) tracking system. Percentage of time spent/distance in open arms were quantified.

### Open field

The open field test was conducted in a square arena (40 cm × 40 cm × 40 cm) with an overhead camera (54). Each mouse was placed in the center of the arena and allowed to freely explore for 5-minutes. The arena was divided into center and outer region using SA215 tracking system (Sansbio, China). Total distance traveled, percentage of distance/time spent in the central zone and number of wall-climbing counts were quantified.

### Barnes maze

A circular platform (diameter 91 cm) containing equally sized 18 holes was mounted on a rotatable stand 80 cm above the ground (51). During the training phase (Days 1–3), an escape box was placed beneath the target hole (fixed for each mouse). Mice were allowed to freely explore the maze until they enter the target hole. If a mouse failed to find the target hole within 3min, it was gently guided to the target hole. During the testing phase (Day 4), all holes were left empty, and the mouse behavior was recorded for 3 minutes and analyzed using SA208 tracking system (Sansbio, China). Latency to first reach the target hole, percentage of time spent/distance traveled around the target hole were quantified. After each trial, the maze was wiped with a chlorine-based disinfectant.

### RNA-seq and data analysis

Three days post CS infection, hippocampal tissues were collected and rapidly preserved in liquid nitrogen, then sent to Novogene (China) for RNA transcriptome sequencing. Raw sequencing reads were first filtered using Fastp software to acquire high quality paired-end clean reads. The clean reads were aligned to the Mus musculus mm39 reference genome using the HISAT2 software to obtain their mapping information on the reference genome (Mapping and quantifying mammalian transcriptomes by RNA-Seq). Reads mapped to the exon regions of each gene were counted by featureCounts (v1.5.0-p3) and FPKM (Fragments Per Kilobase per Millions base pairs) values were calculated for each gene.

Differentially expressed genes (DEGs) between groups were performed using the DESeq2 R package (1.20.0) (Moderated estimation of fold change and dispersion for RNA-seq data with DESeq2). The resulting P-value is adjusted using the Benjamini and Hochberg’s methods to control the error discovery rate. The corrected P-value ≤ 0.05 & |log2 (foldchange)| ≥ 1 was set as the threshold of significant differential expression. Gene Ontology (GO) and Kyoto Encyclopedia of Genes and Genomes (KEGG) enrichment analyses of DEGs were implemented by the clusterProfiler R package(3.8.1), with an adjusted P-value (padj) < 0.05 were considered significantly enriched.

### Statistical analysis

SPSS 21.0 software was used to analyze significant differences. Homogeneity of variance test and normality test was performed for the experimental data. If multiple sets of variables were consistent with homogeneity of variance, analysis of variance was used to compare multigroup variables, and least—significant difference (LSD) test was used to compare intergroup variables. If homogeneity of variance was not assumed, Dunnett’s T3 test was used to compare intergroup variables. Values were represented as the mean ± standard deviation. Values of p < 0.05 were considered significant.

## Results

### HLA DP/DRA expression was elevated in the brain of sepsis patients and HMC3 cells

**Figure 1:**
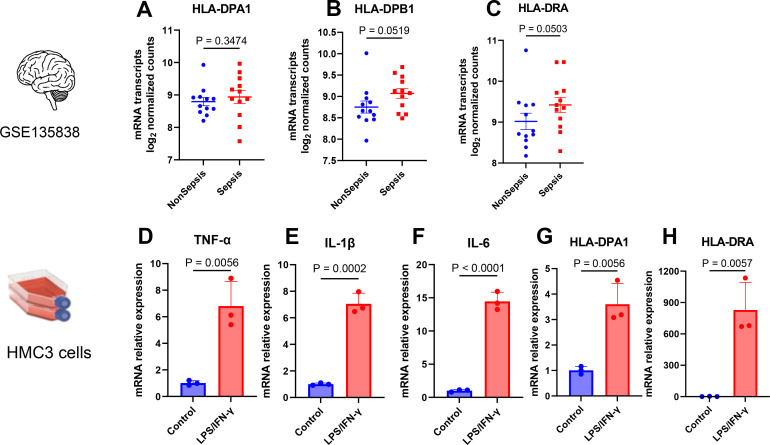
Upregulated expression of HLA DP and DRA in brain tissue of septic patients and HMC3 cell lines. **(A–C)** Analysis of gene expression omnibus (GEO) data from GSE135838. Note a trend toward decreased expression of HLA-DPB1 **(B)** and -DRA **(C)** expression level. **(D)** HMC3 cell lines were stimulated by LPS and IFNγ. Pro-inflammatory cytokines, including TNFα, IL-1β and IL-6, are significantly increased, and the expression level of HLA-DPA1 and -DRA is significantly elevated. Data are represented as mean ± SEM.

To investigate the expression of HLA II molecules (HLA-DP/DRA) in nervous tissue, we analyzed publicly available gene expression omnibus (GEO) data derived from septic patients. Results showed that the mRNA level of HLA-DP and -DR molecules tended to be upregulated in the brains (**Figures 1A–C**). We further stimulated the human microglial clone 3 cell line (HMC3) by lipopolysaccharide (LPS)-and interferon-gamma (IFN-γ) (55, 56). Consistent with previous reports (56, 57), LPS/IFN-γ stimulation rapidly upregulated the expression of tumor necrosis factor-α (TNFα) (**Figure 1D**) and interleukin-1β (IL-1β) (**Figure 1E**), and interleukin-6 (IL-6) (**Figure 1F**) in HMC3 cells. Similarly, the expression of HLA-DPA1 (**Figure 1G**) and HLA-DRA (**Figure 1H**) was also markedly increased in LPS/IFN-γ-stimulated HMC3 cells.

### Introduction of HLA DP/DRA molecules led to increased mortality and severe clinical symptoms in experimental sepsis

Two well-established intraperitoneal sepsis models that closely mimic human sepsis pathophysiology are cecal ligation and puncture (CLP) (58) and peritoneal contamination and infection (PCI) model (59). Compared to CLP model (60), the PCI model is simple, non-surgical, and capable of inducing long-term behavioral impairment (51, 54). To characterize gut microbiota composition, 16S rDNA amplicon sequencing was performed on the cecal contents used in this study (**Supplementary Figure S1A**). We then established the PCI model in C57BL/6J mice via intraperitoneal injection of cecal slurry (CS). The Murine Sepsis Score (MSS) was used to evaluate the sepsis severity (52). Administration of 1.2mg/kg CS resulted in 100% lethal within 48 hours in the absence of antibiotic treatment (**Supplementary Figures S1B, C**), consistent with previous reports (50, 61). To simulate clinical conditions, the potent broad-spectrum antibiotic meropenem was administered to mice at an early (6h) and late (12h) time point after lethal-dose CS injection. Approximately 40% of animals survived when antibiotics were initiated at 6 h, whereas only 10% survived when treatment was delayed to 12 h (**Supplementary Figures S1D, E**). Lethal-dose CS was further administered to HLA-DP401/DRA-IAβ^-/-^ and IAβ^-/-^ mice, with antibiotics treatment at either 6 h or 12 h. IAβ^-/-^ PCI mice exhibited significantly lower sepsis scores at 6 h time point. At 12 h, sepsis scores in IAβ^-/-^ PCI mice remained lower than wild-type (WT) PCI mice. In contrast, sepsis scores in HLA DP401/DRA-IAβ^-/-^ mice were comparable to those in WT mice, suggesting that the loss of murine class II molecules confers resistant to bacterial infection (**Supplementary Figures S1F–I**). For subsequent experiments, a regimen of 1.2 mg/kg CS injection plus antibiotics administration at 6 h was employed.

**Figure 2:**
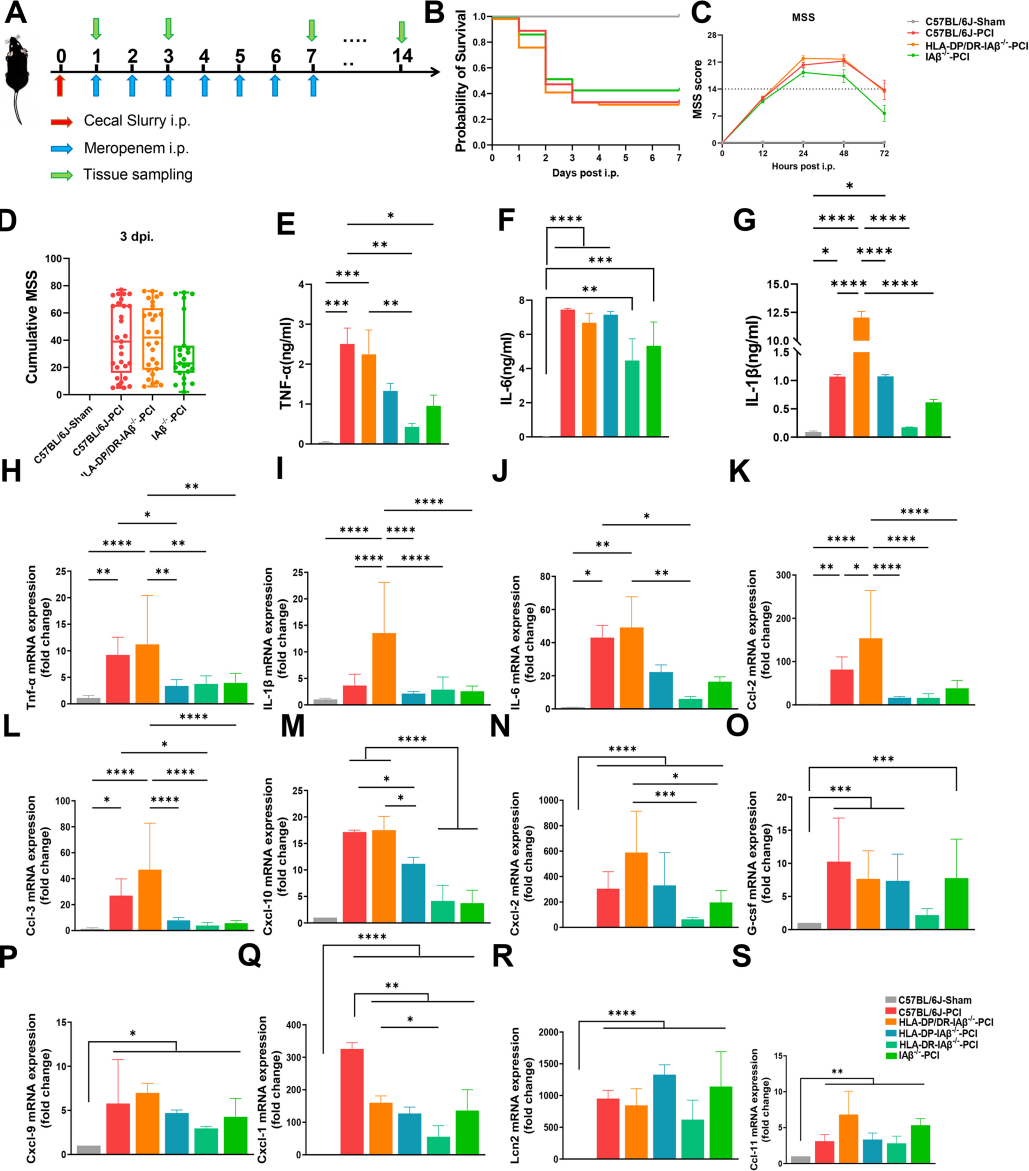
Experimental sepsis induces significant upregulation of cytokines and chemokines in serum and hippocampal tissue. **(A)** Experimental schematic. The acute phase of PCI is marked in red. Antibiotics (meropenem) are delivered intraperitoneally until day 7 (blue arrow). Tissues are collected at the indicated time points (green arrow). **(B)** Kaplan-Meier survival curve of all mice used in PCI experiments, regardless of final experimental readouts. The overall survival rate: C57BL/6J (n = 72, 33%), HLA-DP/DRA-IAβ^-/-^ (n = 63, 32%) and IAβ^-/-^ (n=46, 43%) mice. **(C)** Murine Sepsis Score (MSS) within 3 days. **(D)** The cumulative 3-day MSS was significantly higher in C57BL/6J (42) and HLA-DP/DRA-IAβ^-/-^ (39) PCI mice than in IAβ^-/-^ PCI mice (24.5). The serum concentration of TNF-α **(E)**, IL-6 **(F)**, IL-1β **(G)** measured by ELISA (n = 5). **(H–S)** The hippocampal mRNA expression levels of cytokine and chemokine, including TNFα **(H)**, IL-1β **(I)**, IL-6 **(J)**, CCL-2 **(K)**, CCL-3 **(L)**, CXCL-10 **(M)**, CXCL-2 **(N)**, G-CSF **(O)**, CXCL-9 **(P)**, CXCL-1 **(Q)**, LCN2 **(R)**, CCL-11 **(S)**. Data are represented as mean ± SEM *P < 0.05, **P < 0.01, ***P < 0.001, ****P < 0.000.

We induced severe experimental sepsis in a total of 181 mice via CS injection (**Figure 2A**). The overall survival rate over one week was similar between C57BL/6J (*n* = 72) and HLA-DP/DRA-IAβ^-/-^ (*n* = 63) PCI mice (33% vs. 32%), and both were lower than IAβ^-/-^ (*n* = 46, 43%) PCI mice (**Figures 2B, C**). This survival rate was consistent with that reported for C57BL/6J PCI mice in previous studies (51). C57BL/6J and HLA-DP/DRA-IAβ^-/-^ PCI mice exhibited comparable median cumulative 3-day MSS scores (42 vs. 39), both of, which were significantly higher than that in IAβ^-/-^ (24.5) PCI mice (**Figure 2D**). These results indicate that the loss of endogenous MHC II molecules alleviates acute inflammation following infection, and that the introduction of HLA II molecules (DP and DRA gene) partially reverses the inflammatory protection conferred by endogenous MHC II deficiency. For subsequent experiments, only surviving mice with severe sepsis, defined as a cumulative 3-day MSS above the median value, were included for further analyses.

### Introduction of HLA-DP and HLA-DRA genes enhanced inflammatory cytokine responses in the serum and hippocampus of septic mice

To determine how the introduction of DP and DRA gene modulates systemic and central nervous system inflammation, we employed ELISA and qPCR to measure the expression of inflammatory mediators. ELISA results showed that the serum levels of TNFα and IL-1β were highest in HLA-DP/DRA-IAβ^-/-^ PCI mice among the IAβ^-/-^ knockout genetic background mice at day 1 post-infection (**Figures 2E–G**). Importantly, serum IL-1β concentrations in HLA DP/DRA-IAβ^-/-^ PCI mice were even significantly higher than those in C57BL/6J PCI mice, suggesting that HLA-DP and HLA-DRA synergistically exacerbate systemic inflammation (**Figures 2E–G**). Correspondingly, qPCR analysis revealed that the introduction of HLA-DP and -DRA not only synergistically upregulated mRNA level of TNFα (**Figure 2H**) and IL-1β (**Figure 2I**), but also increased hippocampal expression of the chemokines Ccl-2 (**Figure 2K**), Ccl-3 (**Figure 2L**), Cxcl-10 (**Figure 2M**), and Cxcl-2 (**Figure 2N**) on day 1. In addition, CS injection markedly elevated hippocampal mRNA levels of other inflammatory factors in PCI mice, including Il-6 (**Figure 2J**), G-csf (**Figure 2O**), Cxcl-9 (**Figure 2P**), Cxcl-1 (**Figure 2Q**), Lcn2 (**Figure 2R**), Ccl-11 (**Figure 2S**). These findings are consistent with the previous reports demonstrating concurrent upregulation of multiple cytokine mRNAs in the serum and brain during sepsis (9, 62–66). Collectively, these results indicate that endogenous deficiency of mouse MHC II (IAβ) significantly attenuated post-infection inflammatory response (including cytokine and chemokine expression), and that the introduction of HLA-DP/DRA genes partially reverses this anti-inflammatory effect conferred by MHC class II deficiency.

### Sepsis induced microglia activation and severe hippocampal pathological damage

**Figure 3:**
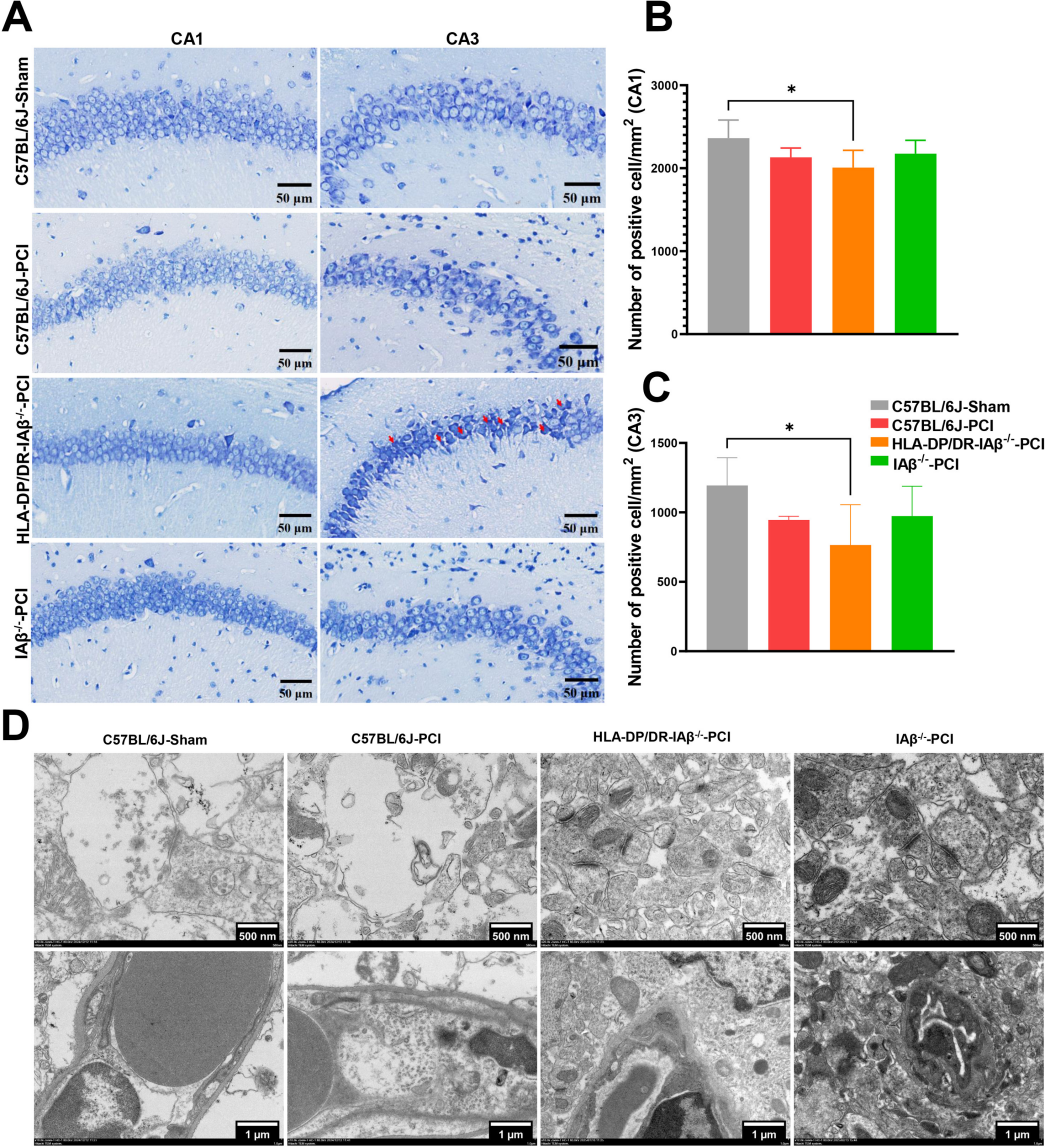
Nissl staining and TEM of hippocampus tissues. **(A)** Representative Nissl staining images of hippocampus tissues at D14. Severe neuronal damage with more obvious cellular morphological abnormalities (arrow) was observed in the CA1 and CA3 regions of the HLA-DP/DRA-IAβ^-/-^ PCI mice at D14. **(B, C)** Reduced number of Nissl bodies in CA1 **(B)** and CA3 **(C)** regions of the HLA-DPDRA-IAβ^-/-^ PCI mice at D14. **(D)** Representative TEM images showing mitochondria (the first line) and microcapillaries (the second line) in the mouse hippocampus.

Published studies have shown that microglia can be activated by inflammatory mediators, adjacent cells and neurotransmitters, which further contributes to neuroinflammation and induces neuronal dysfunctions (8, 54, 67). To evaluate the microglia activation, we performed the immunofluorescence staining with an Iba1 antibody on frozen hippocampal sections (**Figure 3A**). We observed increased microglial soma size (**Figure 3B**) and immunofluorescence intensity (**Figure 3C**) in PCI mice. An increase in the absolute microglial count was also observed in C57BL/6J PCI mice but not in PCI mice with an IAβ^-/-^ background (**Figure 3D**), indicating that endogenous MHC class II deficiency suppresses excessive microglial activation. Consistent with previous studies, the proportion of Iba-1 positive cells remained unchanged (68, 69). In contrast, immunofluorescence intensity of NeuN- and BDNF- positive cells were significantly elevated in all PCI mice (**Supplementary Figure S2A–C**).

**Figure 4:**
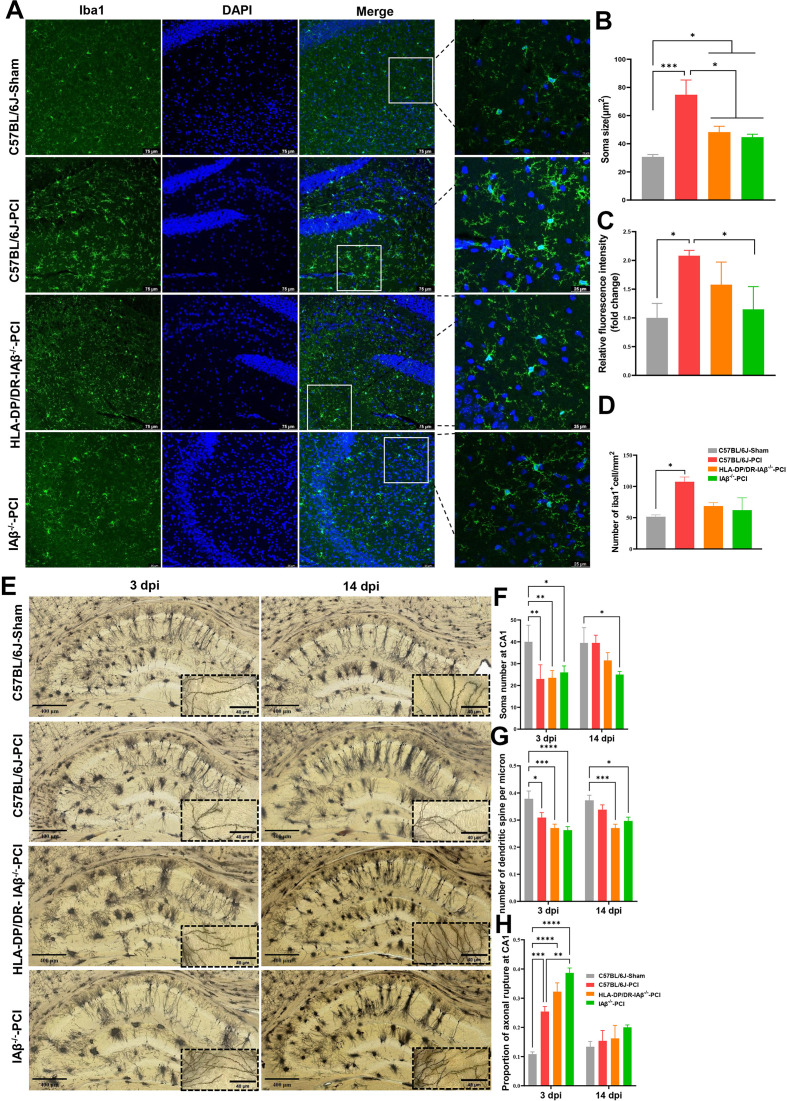
Sepsis induces microglia activation and severe pathological damage in the hippocampus. **(A)** Immunofluorescence staining for Iba1 was performed to evaluate microglial activation. **(B, C)** Increased soma size **(B)** and immunofluorescence intensity **(C)** of microglia in PCI mice. **(D)** An increase of absolute microglia count in C57BL/6J mice, but not in mice with IAβ^-/-^ background. **(E)** Representative Golgi staining images of hippocampal tissues. **(F–H)** Quantification of cell body number **(F)**, dendritic spine density **(G)**, and dendritic axis breakage **(H)** in the CA1 region at D3 and D14. Data are represented as mean ± SEM *P < 0.05, **P < 0.01, ***P < 0.001, ****P < 0.000.

To investigate the pathological changes, we performed Golgi staining (**Figures 3E–H**), Nissl staining (**Figures 4A–C**) and transmission electron microscopy (TEM) (**Figure 4D**) on hippocampal tissues from PCI mice. Golgi staining showed that, compared with control mice, PCI mice exhibited a significant reduction in the number of cell bodies (**Figure 3F**), a marked decrease in the CA1 region on day 3 (D3) (**Figure 3G**), and a significant increase in dendritic axis breakage (**Figure 3H**). Even on day 14 (D14), the number of cell bodies and dendritic spines remained significantly decreased in the CA1 region of PCI mice with IAβ^-/-^ knockout background (**Figures 3F–H**). Nissl staining showed that the neuronal damage remained severe in HLA-DP/DRA-IAβ^-/-^ PCI mice, with more prominent cellular morphological abnormalities and a reduction in Nissl bodies in CA1 (**Figure 4B**) and CA3 (**Figure 4C**) regions on D14. Additionally, TEM analysis revealed significant mitochondrial swelling, mitochondrial cristae rupture, and vacuolar changes in the mitochondrial matrix in PCI mice on D14 (**Figure 4D**).

TEM also revealed an intact microvascular ultrastructure without apparent perivascular edema in the hippocampus of control mice. In WT PCI mice, endothelial cells were significantly swollen and protruded towards the vascular lumen, resulting in luminal stenosis or irregularity (**Figure 4D**). In PCI mice with an IAβ^-/-^ background, the vascular basement membrane exhibited uneven thickness, accompanied by disruption, delamination, and even local dissolution (**Figure 4D**). Collectively, these findings indicate that cecal infection can induce subcellular damage in hippocampal tissue, including neuronal mitochondrial dysfunction and blood-brain barrier disruption.

### Transcriptional profile patterns of the whole hippocampus revealed mitochondrial dysfunction

**Figure 5:**
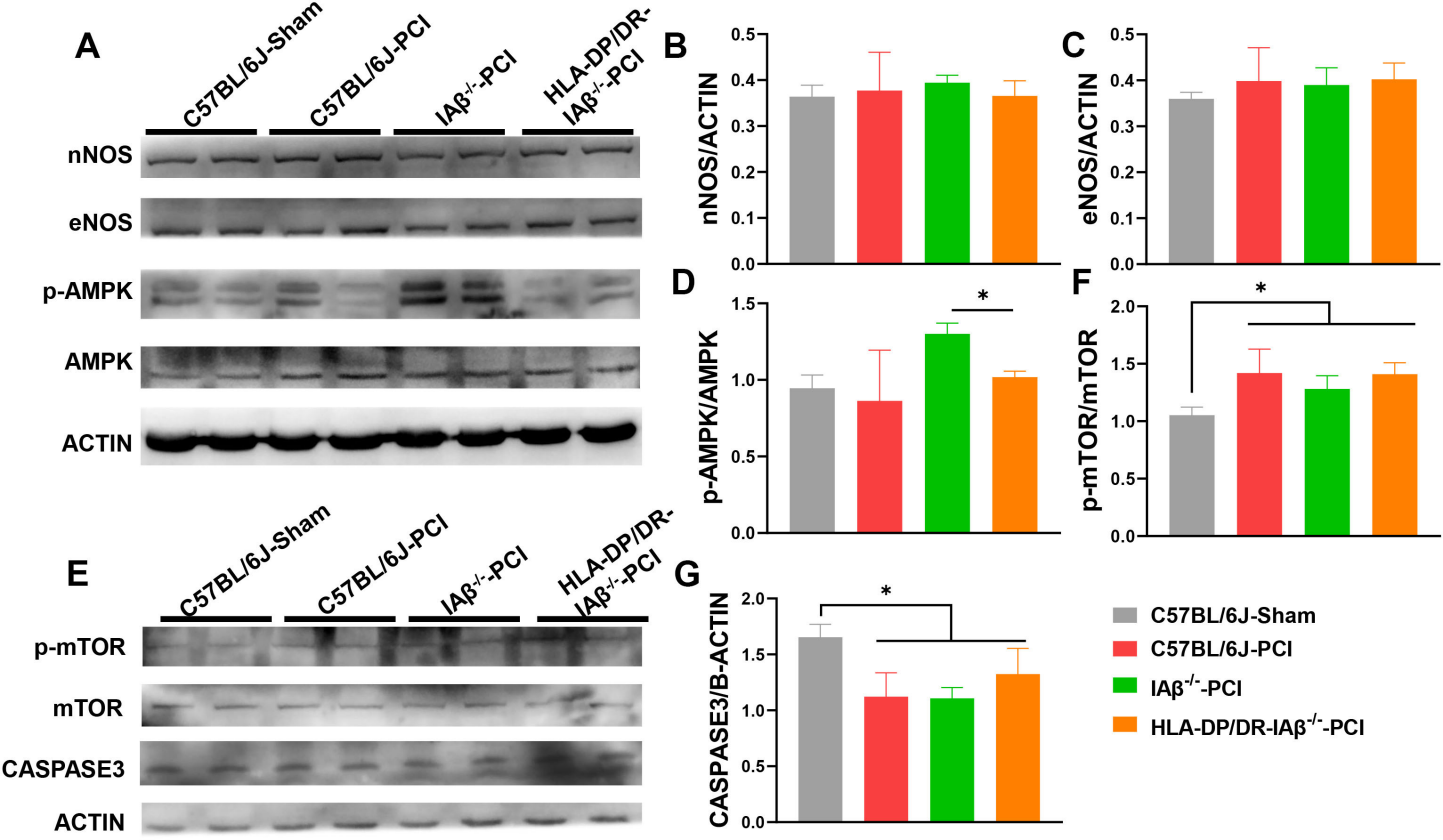
Western blot analysis of AMPK/mTOR signaling pathway in hippocampal tissues from PCI mice. **(A)** Representative western blot and quantitative analysis of nNOS **(B)**, eNOS **(C)**, pAMPK/AMPK **(D)**, β-actin (used as a loading control) (n = 3; *P < 0.05). No significant difference were observed in nNOS or eNOS expression. However, the pAMPK/AMPK ratio was significantly increased in IAβ^-/-^ PCI mice. **(E)** Representative western blot and quantitative analysis of p-mTOR/mTOR **(F)**, cleaved caspase3 **(G)**, and β-actin (used as a loading control) (n = 3, *P < 0.05). A significant increase for the p-mTOR/mTOR ratio in PCI mice. A significant decrease for cleaved caspas3 in PCI mice.

Three days after CS, transcriptome analysis identified a total number of 1829 differentially expressed genes (DEGs) in HLA-DP/DRA-IAβ^-/-^ PCI mice, with 1129 upregulated and 700 downregulated genes (log_2_FC >1 and <−1, respectively). This number was far higher than that in C57BL/6J PCI mice (547 DEGs) and IAβ^-/-^ PCI mice (712 DEGs) (**Figure 5A**; **Supplementary Tables 3–S5**). Compared with IAβ^-/-^ PCI mice, HLA-DP/DRA-IAβ^-/-^ PCI mice had 90 upregulated and 274 downregulated genes, (**Figure 5B**; **Supplementary Table 3**), indicating that HLA transgenes triggers a unique and pronounced reprogramming of gene expression in the IAβ^-/-^ knockout background. Kyoto Encyclopedia of Genes and Genomes (KEGG) pathway analysis revealed enrichment in pathways including” complement and coagulation cascades” and” phagosome” in C57BL/6L PCI mice (**Figure 5C**; **Supplementary Table 4**), consistent with previous reports (54). KEGG pathway analysis revealed the involvement of “oxidative phosphorylation” and “reactive oxygen species” during SAE when compared IAβ^-/-^ with C57BL/6L PCI mice (**Figure 5D**; **Supplementary Table 5**). Reactome analysis further showed enrichment in pathways including “Metabolism of surfactants”, “Erythrocytes take up carbon dioxide”, “ O2/CO2 exchange in erythrocytes”, “degradation of extracellular matrix”, “scavenging of heme from plasma”, demonstrating severe microvascular dysfunction during SAE in IAβ^-/-^ PCI mice (**Supplementary Figure S3A**). when comparing HLA-DP/DRA-IAβ^-/-^ PCI mice with IAβ^-/-^ PCI mice, GO term and KEGG analyses revealed enrichment in categories including “DNA replication dependent nucleosome assembly”, “mitochondrial respiratory chain”, “oxidative phosphorylation”, and “reactive oxygen species” during SAE (**Figure 5E**; **Supplementary Figure S3C**). Reactome analysis identified enrichment pathways including “NoRC negatively regulates rRNA expression”, “SIRT1 negatively regulates rRNA expression”, “RNA polymerase I promoter opening”, “negative epigenetic regulation of rRNA expression”, “activated PKN1 stimulates transcription” (**Supplementary Figure S3B**). These DEGs were visualized in a heatmap (**Figure 5F**). We found downregulation of gene associated with the mitochondrial respiratory chain (e.g., mt-ATP6, mt-ATP8, mt-ATP5g1), oxidative phosphorylation (e.g., mt-ND3, mt-ND4l, mt-COX7b, mt-CO3), and microtubule assembly (e.g., Tubb3, Tubb5, Tubb4b) (**Figure 5F**). STRING network analysis further revealed protein-protein interactions of DEGs related to mitochondrial function in hippocampal tissue (**Figure 5G**), providing a transcriptional basis for the observed pathological change. We also found significant upregulation of genes associated with the mTOR pathway (e.g., mTOR, Prkaa2) (**Supplementary Figure S3D**), and significant downregulation of Hmgb1 (**Supplementary Figure S3D**), indicating that targeting of mTOR signaling pathway could mediate neuroprotection during SAE (70, 71). QPCR analysis confirmed increasing expression of HLA DPA1, DPB1 and DRA in hippocampus from PCI mice (**Supplementary Figures S4A–F**), consistent with previous reports (38, 72, 73) Collectively, these results indicate that HLA-DP/DRA transgenes likely reshaped the post-infection response primarily by modulating mitochondrial energy metabolism and its interactions with immune-related proteins.

### CS treatment upregulated AMPK-α phosphorylation in IAβ^-/-^ mice but not in HLA-DPDRA-IAβ^-/-^ mice

**Figure 6:**
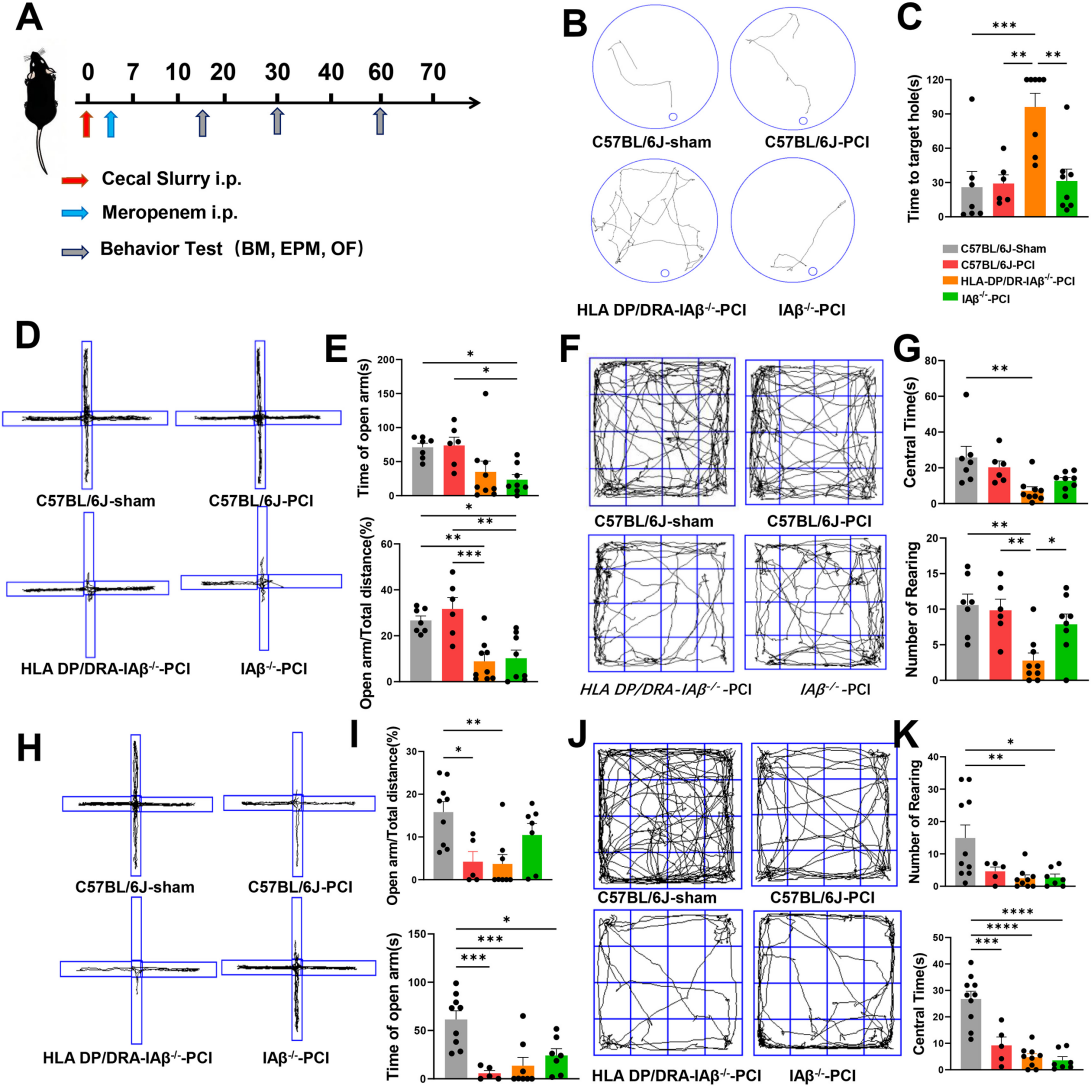
Sepsis induces neurocognitive dysfunction and anxiety in HLA-DP/DRA-IAβ^-/-^ mice at D14, and long-term anxiety-like behavior at D60. **(A)** Experimental timeline. **(B, C)** In the Barnes maze test (BM) at 14 dpi, HLA-DP/DRA-IAβ^-/-^ PCI mice took longer to locate the target hole, indicating impaired spatial memory. **(D, E)** In the elevated plus maze test (EPM) at 14 dpi, mice with an IAβ^-/-^ knockout (HLA-DP/DRA-IAβ^-/-^ and IAβ^-/-^) spent less time and traveled shorter distances in open arms compared with WT-PCI and control mice, indicating increased anxiety-like behavior. **(F, G)** In the open field (OF) at 14 dpi, HLA-DP/DRA-IAβ^-/-^ PCI mice spent less time in the center and exhibited the fewest rearing events. Data are represented as mean ± SEM * *P* < 0.05, ** *P* < 0.01, **(H, I)** In the EPM at 60 dpi, HLA-DP/DRA-IAβ^-/-^ and WT PCI mice traveled shorter distances compared with sham group. PCI mice spend less time in open arms than sham group. **(J, K)** In the OF test at 60 dpi, PCI mice spent less time in the center and exhibited fewer rearing events. Data are represented as mean ± SEM * *P* < 0.05, **P < 0.01, ***P < 0.001, ****P < 0.000. **(F, G)**.

Previous studies have showed that AMPK/mTOR pathways are involved in cognitive impairment during sepsis (74–77). Therefore, we examined whether CS treatment can regulate AMPK/mTOR pathways in hippocampus using western blot analysis (**Figures 6A–G**). As shown in **Figures 6A**, AMPK-α phosphorylation was significantly increased in IAβ^-/-^ PCI mice. In contrast, But AMPK-α phosphorylation level were similar between HLA-DP/DRA-IAβ^-/-^ and WT PCI mice (**Figures 6A, D**), indicating that the introduction of HLA-DP/DRA molecule restored AMPK-α phosphorylation to wild-type levels. mTOR Phosphorylation was significantly elevated in PCI mice (**Figure 6F**), whereas cleaved caspase3 levels were significantly decreased in PCI mice (**Figure 6G**). Downregulation of caspase3 expression post-infection may reflect a compensatory anti-apoptotic response of the organism. Additionally, CS treatment did not significantly alter the expression levels of neuronal nitric oxide synthase (nNOS) (**Figure 6B**) and endothelial nitric oxide synthase (eNOS) (**Figure 6C**).

### Sepsis induced neurocognitive dysfunction and long-term anxiety in HLA-DP/DRA-IAβ^-/-^ PCI mice

**Figure 7:**
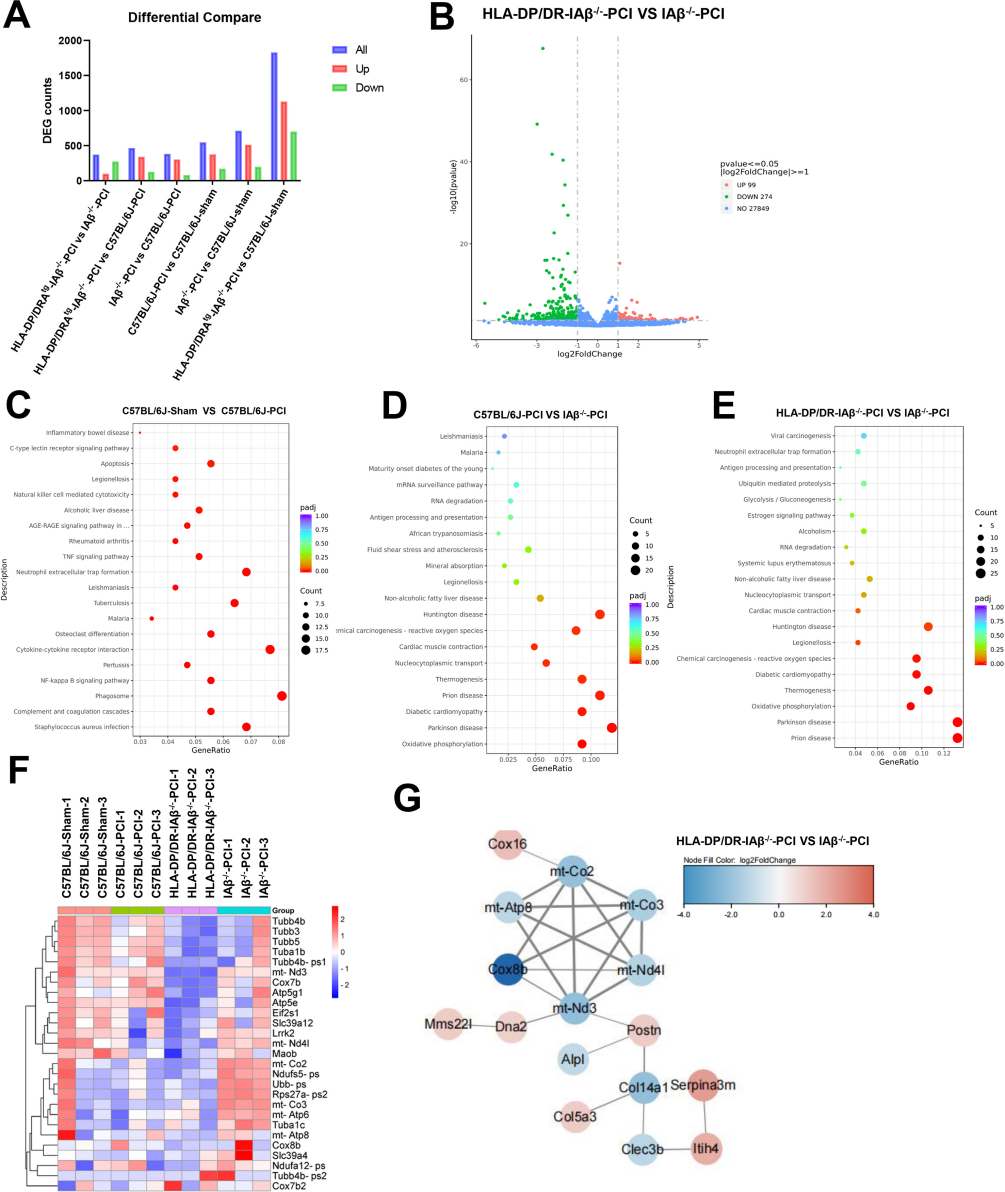
Transcriptional changes in hippocampal tissues during SAE and Kyoto encyclopedia of genes and genomes (KEGG) pathway analysis. **(A)** DEGs in whole hippocampus tissue at day 3 following sepsis induction (n = 3 per group). **(B)** Volcano plot of gene expression in mice with IAβ^-/-^ background. **(C–E)** KEGG pathway enrichment analysis in whole hippocampal tissue at day 3 after sepsis induction: C57BL/6J-PCI vs. C57BL/6J-Sham mice **(C)**, IAβ^-/--^PCI vs. C57BL/6J-PCI mice **(D)**, and HLA-DP/DRA-IAβ^-/-^ PCI vs. IAβ^-/-^ PCI mice **(E)** (n = 3; *P* < 0.05). **(F)** Expression heatmap of hippocampal genes related to mitochondrial respiratory chain, oxidative phosphorylation, and microtubule assembly in PCI- and sham-treated mice at day 3 (n = 3; *P* < 0.05). The color scale represents the gene-wise z-score calculated from normalized gene expression levels. **(G)** STRING network showing protein-protein interactions of DEGs in hippocampal tissue associated with mitochondrial function in hippocampal tissue from PCI- and sham-treated animals at day 3 following sepsis (n = 3; medium-confidence cutoff = 0.4).

Two weeks after CS induction (**Figure 7A**), the Barnes maze (BM) test revealed that HLA -DP/DRA-IAβ^-/-^ PCI mice took longer to locate the target hole (**Figures 7B, C**), indicating impaired spatial memory. The elevated plus maze (EPM) test showed that both HLA-DP/DRA-IAβ^−^/^−^ and IAβ^−^/^−^ PCI mice spent less time and traveled a shorter distance in the open arms compared with WT PCI and sham mice (**Figures 7D, E**), indicating that mice with an IAβ^-/-^ knockout background exhibited pronounced anxiety-like behavior. Furthermore, the open field (OF) test confirmed that HLA-DP/DRA-IAβ^−^/^−^ PCI mice still displayed increased anxiety-like behavior and reduced spatial exploration (**Figures 7F, G**), indicating that the introduction of HLA-DP/DRA gene may exacerbate anxiety-like behavior and impair spatial exploration activity.

Four weeks after CS induction, the least time (**Supplementary Figures S5A, B**) and distance (**Supplementary Figures S5A, C**) of open arm in EPM was found in HLA-DP/DRA-IAβ^-/-^ PCI mice, indicating the existence of a sustained anxiety-like behavior. Moreover, the weakest spatial exploration ability was continuously observed in HLA-DP/DRA-IAβ^-/-^ PCI mice by OF (**Supplementary Figures S5D–S5F**). Defective memory performance revealed by BM have recovered in HLA-DP/DRA-IAβ^-/-^ mice at this time point (**Supplementary Figure S5G**).

Eight weeks after CS induction, time of open arm was significantly decreased in PCI mice, distance of open arm was similar between IAβ^-/-^ PCI mice and sham group, indicating that anxiety-like behavior revealed by EPM was partially alleviated in IAβ^-/-^ PCI mice (**Figures 7H, I**). In addition, we can still observe the increased anxiety-like behavior and weaker spatial exploration ability in all PCI mice (**Figures 7J, K**). Together, these data indicate that CS injection can induce a defective memory performance, long-term anxiety-like behavior and weakened spatial exploration in HLA-DP/DRA-IAβ^-/-^ PCI mice.

## Discussion

Sepsis, a life-threatening organ dysfunction, is triggered by a dysregulated host response to infection caused by bacteria, fungi, viral, and parasitic pathogens. Previous studies have shown that the downregulation of monocytic HLA-DR (mHLA-DR) expression is an early event in sepsis that is strongly associated with both sepsis-induced mortality and an elevated risk of secondary infections. For this reason, mHLA-DR has been proposed as a general biomarker of sepsis-induced immunosuppression (16, 78–80). The class II transactivator (CIITA), a highly conserved regulatory module, controls the transcription of MHC class II genes, including *HLA-DP*, *-DQ*, and *-DR* (81). In humans, a splicing mutation in the CIITA gene leads to transactivator dysfunction, resulting in MHC class II deficiency and severe immunodeficiency known as bare lymphocyte syndrome (31, 82). Single nucleotide polymorphism (SNP) in the *CIITA* promoter can alter the kinetics of mHLA-DR expression, influence disease, severity and mortality in patients with sepsis (32). Integrated transcriptomic and regulatory network analyses have demonstrated that *HLA-DPB1* transcription is reduced in sepsis patients, and that its interplay with miR-let-7b-5p and transcription factor SPIB may play an critical role during sepsis progression (34). Single-cell RNA sequencing and transcriptomic profiling have also identified *HLA DPB1* as one of nine key genes dysregulated in sepsis (33). Moreover, reduced expression of multiple HLA-related genes, including *HLA-DMA*, *HLA-DPA1*, *HLA-DPB1*, *HLA-DRA*, *HLA-DRB1*, and *HLA-DRB5*, has been observed in CHIT1^+^neutrophils (83). However, sufficient evidence remains lacking regarding whether *HLA-DP* and *HLA-DRA* act synergistically during sepsis and contribute to the pathogenesis of sepsis-associated encephalopathy. In this study, we provide direct evidence that the *HLA-DP/DRA* genes modulate sepsis severity. Expression of the *HLA-DP* and *HLA-DRA* genes synergistically upregulate systemic and hippocampal inflammatory cytokines, exacerbates clinical symptoms, impair memory function, and promotes long-term anxiety-like behavior.

Compared with the CLP model, the PCI model is characterized by the absence of surgical trauma and is reliable and easy to perform (59, 84). In particular, recent studies have shown that the PCI model consistently induces long-term cognitive impairment, in contrast to the more transient neurocognitive deficits typically observed after LPS challenge (9, 51, 60). Of note, emerging evidence indicates that CLP models can also produce lasting cognitive dysfunction under specific experimental conditions, including variations in surgical severity, resuscitation strategies, and observational duration (58, 85, 86). Therefore, the distinction between models is not absolute, and the duration and severity of cognitive impairment vary according to the specific experimental design employed.

The PCI model contain a mixture of various aerobic and anaerobic Gram-positive and Gram-negative bacteria (87). Because of the lower binding affinity of SAgs for murine MHC class II as compared with human HLA class II, conventional mice are much less sensitive to SAg-mediated effects than humans (44, 88, 89). We were particularly interested in HLA-tg mice carrying the HLA class II alleles associated with high-risk for sepsis, as identified in previous epidemiological and bioinformatics studies (16, 33, 34). The humanized mice used in this study, as previously characterized as HLA DP401/DRA01 transgenic mice, preserves the MHC II haplotype context using a bacterial artificial chromosome (BAC) containing both the *HLA-DP/DRA* genes and their native flanking and intervening sequences (45, 47). Single-chain DRα Tg systems have revealed interspecies pairing between I-E and HLA-DR in IAβ^-/-^ mice. DPα/DPβ, DRα/Eβ as well as Eα/Eβ molecules act as MHC class II molecules to shape the T-cell receptor repertoire and stimulate the mixed lymphocyte reaction (90). In these mice, most class II restriction elements are of human origin, thereby partially eliminating interspecies pairing between the class II chains and allowing us to explore the role of MHC class II molecules in infectious diseases (45).

This study, through the triangulation of clinical, cellular, and animal experiments, established HLA-DP/DRA as a central regulatory hub in septic neuroinflammation. Analysis of the GSE135838 dataset revealed an upregulation trend in mRNA expression of *HLA-DPA1*, *HLA-DPB1*, and *HLA-DRA* in the brain tissue of sepsis patients (*HLA-DPB1* P = 0.0519, *HLA-DRA* P = 0.0503). This finding suggests a direct correlation between the expression dynamics of HLA-DP/DRA and human septic brain injury, filling the knowledge gap regarding the association of HLA molecules with septic neuroinflammation at the clinical level. Experiments using HMC3 microglia confirmed that LPS/IFN-γcan directly induce the upregulation of HLA-DPA1 (P = 0.0056) and HLA-DRA (P = 0.0507) expression, which is synchronized with the activation of pro-inflammatory cytokines (TNF-α, IL-1β, IL-6). Those results identify microglia as the primary effector cells of HLA-DP/DRA in the central inflammatory response. Their upregulated expression enhances antigen-presenting capacity, forming a positive feedback loop of “inflammation to HLA expression to amplified inflammation”, thereby providing a molecular basis for the sustained exacerbation of neuroinflammation. In the HLA-DP/DR-IAβ^-/-^ double-transgenic mouse model, HLA-DP/DRA expression significantly exacerbated microglial overactivation, blood-brain barrier disruption, and neuronal damage. This ultimately led to persistent long-term impairments, including spatial memory deficits, anxiety-like behaviors, and reduced exploratory behavior. These outcomes directly validate the inferences drawn from the clinical and cellular experiments, establishing a causal link between HLA-DP/DRA expression and behavioral deficits.

This study also broadens the conventional understanding of septic neuroinflammation by demonstrating how HLA-DP/DRA drives behavioral impairment. HLA-DP and HLA-DRA synergistically upregulated systemic inflammation and neuroinflammation. Specifically, the release of these cytokines and chemokines has been shown to play a critical role in the development of long-lasting neuroinflammation following the initial sepsis insult (9, 62, 91). Markedly elevated peripheral inflammatory cytokines, including IL-1β and TNFα, transmit signals across the impaired blood-brain barrier (BBB) and activate microglia (9). Peripheral inflammatory cells, including NK cells, infiltrate into the CNS during early sepsis (12). HLA-DP401 may interact with NK cells within the CNS. These activated NK cells further modulate microglia to recruit neutrophils, and upregulate the mRNA level of cytokines and chemokines including Ccl-2, Ccl-3, Cxcl-10 and Cxcl-2, ultimately leading to cognitive dysfunction and long-term anxiety-like behavior.

Adenosine monophosphate−activated protein kinase (AMPK) is a major energy sensor that regulates an array of downstream target genes and maintains cellular energy homeostasis (92). Previous studies in neurodegenerative disorders and sepsis have demonstrated that AMPK activation can exert either neuroprotective or deleterious effects in distinct neuronal subtypes, depending on the pathological context. This inconsistency may be attributable to differences in the specific animal models, species, sex, or route of administration used (74, 93, 94). Using a classical sepsis model, we found that AMPK activity was increased in IAβ^^−^/^−^^ PCI mice, whereas AMPK activity was comparable between HLA−DP/DRA−IAβ^^−^/^−^^ and WT PCI mice. Compared with IAβ^^−^/^−^^ PCI mice, mitochondrial dysfunction and reduced pAMPK levels may contribute to memory impairment in HLA−DP/DRA−IAβ^^−^/^−^^ PCI mice. AMPK activity in the CA1 region negatively regulates contextual fear memory formation and structural plasticity via mTORC1 signaling (77). The mechanisms by which introduction of the HLA−DP/DRA molecule restores pAMPK levels warrant further investigation.

In short, these findings highlight the dominant role of HLA class II in determining the outcome of SAE by modulating the systemic and local inflammatory cytokine responses to bacterial infection. These results from the HLA-transgenic mice parallel bioinformatics findings in humans and confirm a direct and dominant role of HLA class II alleles (HLA-DP/DRA) in controlling the magnitude of immune response to sepsis. The development of the HLA-DP/DRA-IAβ^-/-^ mice provides a tractable animal model for studying SAE pathogenesis, with sensitivity to bacterial antigens comparable to that in humans. These findings will advance our understanding of the temporal events underlying SAE pathogenesis, and facilitate the identification of molecular and cellular mechanisms regulating immune responses in the context of distinct HLA class II alleles *in vivo*. Furthermore, HLA-DP/DRA-IAβ^-/-^ mice represent a promising models for SAE, enabling therapeutic drugs screening and efficacy assessment, and exploration of infection mechanisms.

## Data Availability

The data presented in the study are deposited in the Genome Sequence Archive (95) in National Genomics Data Center (96), China National Center for Bioinformation /Beijing Institute of Genomics, accession number CRA029059 (Chinese Academy of Sciences), that are publicly accessible at https://ngdc.cncb.ac.cn/gsa.

## Supplementary material

Supplementary Figure 1 **(A)** Relative abundances of bacteria in the cecal slurry used in this study at the phylum and genus levels. At the phylum level, Firmicutes and Bacteroidota collectively accounted for the majority of total abundance (98%). At the genus level, high relative abundances were observed for *alistipes*, *lachnospiraceae_NK4A136_group*, *eubacterium_siraeum group*, *prevotellaceae_UCG-001*, *colidextribacter, desulfovibrio*, *oscillibacter and turicibacter*. **(B)** Higher doses of CS slurry (CS; 1.25 and 1.5mg/kg) induced higher Murine Sepsis Score (MSS) **(B)**. **(C)** Corresponding survival rate. **(D, E)** Animals received antibiotic treatment (meropenem, 20 mg/kg i.p.) starting at 6 and 12 h after 1.2 mg/kg CS injection; antibiotics were administered twice daily for 5 consecutive days (n = 8–10 per group). MSS **(D)** and survival rate **(E)** were monitored daily. **(F–I)** HLA-DP401/DRA-IAβ^-/-^ and IAβ^-/-^ mice received a lethal dose of CS (1.2 mg/kg) with antibiotic initiation at 6 h or 12 h post-injection. **(F)** mice with IAβ^-/-^ background exhibited significantly lower MSS at the 6 h time point. **(G)** HLA-DP401/DRA-IAβ^-/-^ and WT PCI mice showed higher MSS at the 12 h time point compared with IAβ^-/-^ PCI mice.

Supplementary Figure 2 Sepsis induce the rising expression of NeuN and BDNF in hippocampus from PCI mice. **(A)** Representative images of immunofluorescence staining of NeuN (red), BDNF (green), DAPI (blue) and colocalization in the hippocampus, scale bar = 50 μm. Quantification of the number of NeuN^+^ **(B)** and BDNF^+^ **(C)** cells in the hippocampus among the four groups. Data are presented as the mean ± SEM (n = 3 mice/group). ***P* < 0.01 vs. the indicated groups.

Supplementary Figure 3 Recktome and GO enrichment analysis. Rectome analysis for IAβ^-/-^ PCI vs. C57BL/6J PCI mice **(A)**, HLA-DP/DRA-IAβ^-/-^ versus IAβ^-/-^ PCI mice **(B)** in whole hippocampus tissue at day 3 following sepsis induction (n = 3, *P* < 0.05). **(C)** GO enrichment analysis for HLA-DP/DRA-IAβ^-/-^ PCI mice versus IAβ^-/-^ PCI mice in whole hippocampus tissue at day 3 following sepsis induction (n = 3, *P* < 0.05). **(D)** Expression of DEGs associated to mTOR, AMPK pathway after PCI- and sham-treated mice at day 3 following sepsis induction (n = 3, *P* < 0.05).

Supplementary Figure 4 Measurement of HLA-DP and -DRA mRNA levels in hippocampus tissues from HLA-DP/DRA-IAβ^-/-^ PCI mice. The indicated tissue homogenates were used to measure the expression levels of the HLA-DPA1, -DPB1 and -DRA molecules by QPCR at D1 **(A–C)** and D3 **(D–F)** after CS injection. Note the increasing expression of HLA-DPB1 **(E)** and -DRA **(F)** in hippocampus at D3. Data was shown as the mean ± SEM of three individual mice. ^*^*P* < 0.05.

Supplementary Figure 5 Long-term anxiety persists in septic mice at D30 **(A–C)** In EPM, PCI mice spent less time and traveled fewer distance in open arms, compared with that in control mice, indicating that PCI mice had increased anxiety-like behavior. **(D–F)** In OF, PCI mice had lesser central time and had fewer rearing numbers. **(G)** In BM, PCI mice spent similar time to get the target hole with control mice, indicating a recovery of memory performance. Data are represented as mean ± SEM **P* < 0.05, ***P* < 0.01, ****P* < 0.001, *****P* < 0.000.

## References

[1] Gofton TE Young GB . Sepsis-associated encephalopathy. Nat Rev Neurol. (2012) 8:557–66. doi: 10.1038/nrneurol.2012.183 22986430

[2] Chen J Shi X Diao M Jin G Zhu Y Hu W . A retrospective study of sepsis-associated encephalopathy: epidemiology, clinical features and adverse outcomes. BMC Emerg Med. (2020) 20:77. doi: 10.1186/s12873-020-00374-3 33023479 PMC7539509

[3] Feng Q Ai YH Gong H Wu L Ai ML Deng SY . Characterization of sepsis and sepsis-associated encephalopathy. J Intensive Care Med. (2019) 34:938–45. doi: 10.1177/0885066617719750 28718340

[4] Li J Jia Q Yang L Wu Y Peng Y Du L . Sepsis-associated encephalopathy: mechanisms, diagnosis, and treatments update. Int J Biol Sci. (2025) 21:3214–28. doi: 10.7150/ijbs.102234 40384873 PMC12080397

[5] Sonneville R Benghanem S Jeantin L de Montmollin E Doman M Gaudemer A . The spectrum of sepsis-associated encephalopathy: a clinical perspective. Crit Care. (2023) 27:386. doi: 10.1186/s13054-023-04655-8 37798769 PMC10552444

[6] Barlow B Ponnaluri S Barlow A Roth W . Targeting the gut microbiome in the management of sepsis-associated encephalopathy. Front Neurol. (2022) 13:999035. doi: 10.3389/fneur.2022.999035 36247756 PMC9557965

[7] Hong Y Chen P Gao J Lin Y Chen L Shang X . Sepsis-associated encephalopathy: from pathophysiology to clinical management. Int Immunopharmacol. (2023) 124:110800. doi: 10.1016/j.intimp.2023.110800 37619410

[8] Catarina AV Branchini G Bettoni L De Oliveira JR Nunes FB . Sepsis-associated encephalopathy: from pathophysiology to progress in experimental studies. Mol Neurobiol. (2021) 58:2770–79. doi: 10.1007/s12035-021-02303-2 33495934

[9] Gao S Jiang Y Chen Z Zhao X Gu J Wu H . Metabolic reprogramming of microglia in sepsis-associated encephalopathy: insights from neuroinflammation. Curr Neuropharmacol. (2023) 21:1992–2005. doi: 10.2174/1570159X21666221216162606 36529923 PMC10514522

[10] Xin Y Tian M Deng S Li J Yang M Gao J . The key drivers of brain injury by systemic inflammatory responses after sepsis: microglia and neuroinflammation. Mol Neurobiol. (2023) 60:1369–90. doi: 10.1007/s12035-022-03148-z 36445634 PMC9899199

[11] Dumbuya JS Li S Liang L Zeng Q . Paediatric sepsis-associated encephalopathy (SAE): a comprehensive review. Mol Med. (2023) 29:27. doi: 10.1186/s10020-023-00621-w 36823611 PMC9951490

[12] He H Geng T Chen P Wang M Hu J Kang L . NK cells promote neutrophil recruitment in the brain during sepsis-induced neuroinflammation. Sci Rep. (2016) 6:27711. doi: 10.1038/srep27711 27270556 PMC4897692

[13] Matzaraki V Kumar V Wijmenga C Zhernakova A . The MHC locus and genetic susceptibility to autoimmune and infectious diseases. Genome Biol. (2017) 18:76. doi: 10.1186/s13059-017-1207-1 28449694 PMC5406920

[14] Blackwell JM Jamieson SE Burgner D . HLA and infectious diseases. Clin Microbiol Rev. (2009) 22:370–85. doi: 10.1128/CMR.00048-08 19366919 PMC2668228

[15] Varney MD Valdes AM Carlson JA Noble JA Tait BD Bonella P . HLA DPA1, DPB1 alleles and haplotypes contribute to the risk associated with type 1 diabetes: analysis of the type 1 diabetes genetics consortium families. Diabetes. (2010) 59:2055–62. doi: 10.2337/db09-0680 20424227 PMC2911060

[16] Zhuang Y Peng H Chen Y Zhou S Chen Y . Dynamic monitoring of monocyte HLA-DR expression for the diagnosis, prognosis, and prediction of sepsis. Front Biosci (Landmark Ed). (2017) 22:1344–54. doi: 10.2741/4547 28199206

[17] Heuberger C Pott J Maloy KJ . Why do intestinal epithelial cells express MHC class II? Immunology. (2021) 162:357–67. doi: 10.1111/imm.13270 32966619 PMC7968399

[18] Bar F Sina C Hundorfean G Pagel R Lehnert H Fellermann K . Inflammatory bowel diseases influence major histocompatibility complex class I (MHC I) and II compartments in intestinal epithelial cells. Clin Exp Immunol. (2013) 172:280–89. doi: 10.1111/cei.12047 23574324 PMC3628330

[19] Stevanovic S van Bergen CA van Luxemburg-Heijs SA van der Zouwen B Jordanova ES Kruisselbrink AB . HLA class II upregulation during viral infection leads to HLA-DP-directed graft-versus-host disease after CD4+ donor lymphocyte infusion. Blood. (2013) 122:1963–73. doi: 10.1182/blood-2012-12-470872 23777765

[20] Zhou Y Luo Z Liao C Cao R Hussain Z Wang J . MHC class II in renal tubules plays an essential role in renal fibrosis. Cell Mol Immunol. (2021) 18:2530–40. doi: 10.1038/s41423-021-00763-z 34556823 PMC8545940

[21] Petersdorf EW Malkki M O'HUigin C Carrington M Gooley T Haagenson MD . High HLA-DP expression and graft-versus-host disease. N Engl J Med. (2015) 373:599–609. doi: 10.1056/NEJMoa1500140 26267621 PMC4560117

[22] Guo X Zhang Y Li J Ma J Wei Z Tan W . Strong influence of human leukocyte antigen (HLA)-DP gene variants on development of persistent chronic hepatitis B virus carriers in the Han Chinese population. Hepatology. (2011) 53:422–28. doi: 10.1002/hep.24048 21274863 PMC3056070

[23] Jiang X Ma Y Cui W Li MD . Association of variants in HLA-DP on chromosome 6 with chronic hepatitis B virus infection and related phenotypes. Amino Acids. (2014) 46:1819–26. doi: 10.1007/s00726-014-1767-2 24846544

[24] Mizushima D Hayashida T Nguyen D Nguyen DT Matsumoto S Tanuma J . Possible association of HLA-DP polymorphism and antiretroviral therapy with hepatitis B virus clearance in an HIV-infected Vietnamese population. Glob Health Med. (2022) 4:146–51. doi: 10.35772/ghm.2021.01113 35855066 PMC9243410

[25] Ruggiero A De Spiegelaere W Cozzi-Lepri A Kiselinova M Pollakis G Beloukas A . During stably suppressive antiretroviral therapy integrated HIV-1 DNA load in peripheral blood is associated with the frequency of CD8 cells expressing HLA-DR/DP/DQ. Ebiomedicine. (2015) 2:1153–59. doi: 10.1016/j.ebiom.2015.07.025 26498496 PMC4588402

[26] Amicosante M Berretta F Dweik R Saltini C . Role of high-affinity HLA-DP specific CLIP-derived peptides in beryllium binding to the HLA-DPGlu69 berylliosis-associated molecules and presentation to beryllium-sensitized T cells. Immunology. (2009) 128:e462–70. doi: 10.1111/j.1365-2567.2008.03000.x 19191908 PMC2753961

[27] Niehrs A Garcia-Beltran WF Norman PJ Watson GM Holzemer A Chapel A . A subset of HLA-DP molecules serve as ligands for the natural cytotoxicity receptor NKp44. Nat Immunol. (2019) 20:1129–37. doi: 10.1038/s41590-019-0448-4 31358998 PMC8370669

[28] Baumdick ME Niehrs A Degenhardt F Schwerk M Hinrichs O Jordan-Paiz A . HLA-DP on epithelial cells enables tissue damage by NKp44(+) natural killer cells in ulcerative colitis. Gastroenterology. (2023) 165:946–62. doi: 10.1053/j.gastro.2023.06.034 37454979 PMC10529779

[29] Padoan B Casar C Krause J Schultheiss C Baumdick ME Niehrs A . NKp44/HLA-DP-dependent regulation of CD8 effector T cells by NK cells. Cell Rep. (2024) 43:114089. doi: 10.1016/j.celrep.2024.114089 38615318 PMC11416720

[30] May J Kremsner PG Milovanovic D Schnittger L Loliger CC Bienzle U . HLA-DP control of human Schistosoma haematobium infection. Am J Trop Med Hyg. (1998) 59:302–06. doi: 10.4269/ajtmh.1998.59.302 9715951

[31] Mach B Steimle V Reith W . MHC class II-deficient combined immunodeficiency: a disease of gene regulation. Immunol Rev. (1994) 138:207–21. doi: 10.1111/j.1600-065x.1994.tb00853.x 8070816

[32] Miatello J Lukaszewicz AC Carter MJ Faivre V Hua S Martinet KZ . CIITA promoter polymorphism impairs monocytes HLA-DR expression in patients with septic shock. Iscience. (2022) 25:105291. doi: 10.1016/j.isci.2022.105291 36304101 PMC9593818

[33] Mo Q Mo Q Mo F . Single-cell RNA sequencing and transcriptomic analysis reveal key genes and regulatory mechanisms in sepsis. Biotechnol Genet Eng Rev. (2024) 40:1636–58. doi: 10.1080/02648725.2023.2196475 37017187

[34] Mohsin M Singh P Khan S Verma AK Jha R Alsahli MA . Integrated transcriptomic and regulatory network analyses uncovers the role of let-7b-5p, SPIB, and HLA-DPB1 in sepsis. Sci Rep. (2022) 12:11963. doi: 10.1038/s41598-022-16183-6 35831411 PMC9279366

[35] Siegler BH Thon JN Altvater M Schenz J Larmann J Weigand MA . Abdominal surgery induces long-lasting changes in expression and binding of CTCF with impact on major histocompatibility complex II transcription in circulating human monocytes. PloS One. (2023) 18:e293347. doi: 10.1371/journal.pone.0293347 37878653 PMC10599505

[36] Siegler BH Altvater M Thon JN Neuhaus C Arens C Uhle F . Postoperative abdominal sepsis induces selective and persistent changes in CTCF binding within the MHC-II region of human monocytes. PloS One. (2021) 16:e250818. doi: 10.1371/journal.pone.0250818 33939725 PMC8092803

[37] Pazmany T Mechtler L Tomasi TB Kosa JP Turoczi A Urbanyi Z . Differential regulation of major histocompatibility complex class II expression and nitric oxide release by beta-amyloid in rat astrocyte and microglia. Brain Res. (1999) 835:213–23. doi: 10.1016/s0006-8993(99)01583-8 10415376

[38] Hendrickx D van Eden CG Schuurman KG Hamann J Huitinga I . Staining of HLA-DR, Iba1 and CD68 in human microglia reveals partially overlapping expression depending on cellular morphology and pathology. J Neuroimmunol. (2017) 309:12–22. doi: 10.1016/j.jneuroim.2017.04.007 28601280

[39] Steiner J Mawrin C Ziegeler A Bielau H Ullrich O Bernstein HG . Distribution of HLA-DR-positive microglia in schizophrenia reflects impaired cerebral lateralization. Acta Neuropathol. (2006) 112:305–16. doi: 10.1007/s00401-006-0090-8 16783554

[40] Williams GP Schonhoff AM Jurkuvenaite A Thome AD Standaert DG Harms AS . Targeting of the class II transactivator attenuates inflammation and neurodegeneration in an alpha-synuclein model of Parkinson's disease. J Neuroinflamm. (2018) 15:244. doi: 10.1186/s12974-018-1286-2 30165873 PMC6117927

[41] Rajagopalan G Polich G Sen MM Singh M Epstein BE Lytle AK . Evaluating the role of HLA-DQ polymorphisms on immune response to bacterial superantigens using transgenic mice. Tissue Antigens. (2008) 71:135–45. doi: 10.1111/j.1399-0039.2007.00986.x 18086265

[42] Nooh MM El-Gengehi N Kansal R David CS Kotb M . HLA transgenic mice provide evidence for a direct and dominant role of HLA class II variation in modulating the severity of streptococcal sepsis. J Immunol. (2007) 178:3076–83. doi: 10.4049/jimmunol.178.5.3076 17312154

[43] Rajagopalan G Iijima K Singh M Kita H Patel R David CS . Intranasal exposure to bacterial superantigens induces airway inflammation in HLA class II transgenic mice. Infect Immun. (2006) 74:1284–96. doi: 10.1128/IAI.74.2.1284-1296.2006 16428778 PMC1360368

[44] Tuffs SW Haeryfar S McCormick JK . Manipulation of innate and adaptive immunity by staphylococcal superantigens. Pathogens. (2018) 7(2):53. doi: 10.3390/pathogens7020053 29843476 PMC6027230

[45] Li F Niu B Liu L Zhu M Yang H Qin B . Characterization of genetic humanized mice with transgenic HLA DP401 or DRA but deficient in endogenous murine MHC class II genes upon Staphylococcus aureus pneumonia. Anim Model Exp Med. (2023) 6:585–97. doi: 10.1002/ame2.12331 37246733 PMC10757210

[46] Ni C Han Y Wang Y Ma T Sha D Xu Y . Human HLA prolongs the host inflammatory response in Streptococcus suis serotype 2 infection compared to mouse H2 molecules. Front Cell Infect Microbiol. (2023) 13:1285055. doi: 10.3389/fcimb.2023.1285055 38035330 PMC10682707

[47] Li F Zhu MM Niu BW Liu LL Peng XH Yang H . Generation and expression analysis of BAC humanized mice carrying HLA-DP401 haplotype. Anim Model Exp Med. (2021) 4:116–28. doi: 10.1002/ame2.12158 34179719 PMC8212823

[48] Castelli FA Buhot C Sanson A Zarour H Pouvelle-Moratille S Nonn C . HLA-DP4, the most frequent HLA II molecule, defines a new supertype of peptide-binding specificity. J Immunol. (2002) 169:6928–34. doi: 10.4049/jimmunol.169.12.6928 12471126

[49] Sidney J Steen A Moore C Ngo S Chung J Peters B . Five HLA-DP molecules frequently expressed in the worldwide human population share a common HLA supertypic binding specificity. J Immunol. (2010) 184:2492–503. doi: 10.4049/jimmunol.0903655 20139279 PMC2935290

[50] Rincon JC Efron PA Moldawer LL Larson SD . Cecal slurry injection in neonatal and adult mice. Methods Mol Biol. (2021) 2321:27–41. doi: 10.1007/978-1-0716-1488-4_4 34048005 PMC8482797

[51] Grunewald B Wickel J Hahn N Rahmati V Rupp H Chung HY . Targeted rescue of synaptic plasticity improves cognitive decline in sepsis-associated encephalopathy. Mol Ther. (2024) 32:2113–29. doi: 10.1016/j.ymthe.2024.05.001 38788710 PMC11286813

[52] Shrum B Anantha RV Xu SX Donnelly M Haeryfar SM McCormick JK . A robust scoring system to evaluate sepsis severity in an animal model. BMC Res Notes. (2014) 7:233. doi: 10.1186/1756-0500-7-233 24725742 PMC4022086

[53] Peng Q Liu Y Yu L Shen Y Li F Feng S . Deletion of Arrb2 down-regulates autophagy in the mouse hippocampus via Akt-mTOR pathway activation. Neuroscience. (2023) 519:120–30. doi: 10.1016/j.neuroscience.2023.01.024 36796753

[54] Chung HY Wickel J Hahn N Mein N Schwarzbrunn M Koch P . Microglia mediate neurocognitive deficits by eliminating C1q-tagged synapses in sepsis-associated encephalopathy. Sci Adv. (2023) 9:q7806. doi: 10.1126/sciadv.abq7806 37235660 PMC10219600

[55] Dello RC Cappoli N Coletta I Mezzogori D Paciello F Pozzoli G . The human microglial HMC3 cell line: where do we stand? A systematic literature review. J Neuroinflamm. (2018) 15:259. doi: 10.1186/s12974-018-1288-0 30200996 PMC6131758

[56] Gunasegaran B Krishnamurthy S Chow SS Villanueva MD Guller A Ahn SB . Comparative analysis of HMC3 and C20 microglial cell lines reveals differential myeloid characteristics and responses to immune stimuli. Immunology. (2025) 175:84–102. doi: 10.1111/imm.13900 39961658 PMC11982601

[57] Zhang M Xu Y Zhu G Zeng Q Gao R Qiu J . Human C15orf39 inhibits inflammatory response via PRMT2 in human microglial HMC3 cell line. Int J Mol Sci. (2024) 25(11):6025. doi: 10.3390/ijms25116025 38892217 PMC11173073

[58] Rittirsch D Huber-Lang MS Flierl MA Ward PA . Immunodesign of experimental sepsis by cecal ligation and puncture. Nat Protoc. (2009) 4:31–6. doi: 10.1038/nprot.2008.214 19131954 PMC2754226

[59] Delfrate G Albino LB Assreuy J Fernandes D . Cecal slurry as an alternative model to cecal ligation and puncture for the study of sepsis-induced cardiovascular dysfunction. Shock. (2024) 62:547–55. doi: 10.1097/SHK.0000000000002412 38888572

[60] Savi FF de Oliveira A de Medeiros GF Bozza FA Michels M Sharshar T . What animal models can tell us about long-term cognitive dysfunction following sepsis: a systematic review. Neurosci Biobehav Rev. (2021) 124:386–404. doi: 10.1016/j.neubiorev.2020.12.005 33309906

[61] Steele AM Starr ME Saito H . Late therapeutic intervention with antibiotics and fluid resuscitation allows for a prolonged disease course with high survival in a severe murine model of sepsis. Shock. (2017) 47:726–34. doi: 10.1097/SHK.0000000000000799 27879561 PMC5432399

[62] Hasegawa-Ishii S Inaba M Shimada A . Widespread time-dependent changes in tissue cytokine concentrations in brain regions during the acute phase of endotoxemia in mice. Neurotoxicology. (2020) 76:67–74. doi: 10.1016/j.neuro.2019.10.006 31628962

[63] Guo C Li W Liu Y Mahaman YA Wang J Liu R . LCN2 induces neuronal loss and facilitates sepsis-associated cognitive impairments. Cell Death Dis. (2025) 16:146. doi: 10.1038/s41419-025-07469-4 40025014 PMC11873032

[64] Seemann S Zohles F Lupp A . Comprehensive comparison of three different animal models for systemic inflammation. J BioMed Sci. (2017) 24:60. doi: 10.1186/s12929-017-0370-8 28836970 PMC5569462

[65] Silverman HA Dancho M Regnier-Golanov A Nasim M Ochani M Olofsson PS . Brain region-specific alterations in the gene expression of cytokines, immune cell markers and cholinergic system components during peripheral endotoxin-induced inflammation. Mol Med. (2015) 20:601–11. doi: 10.2119/molmed.2014.00147 25299421 PMC4365063

[66] Gentile LF Nacionales DC Lopez MC Vanzant E Cuenca A Szpila BE . Host responses to sepsis vary in different low-lethality murine models. PloS One. (2014) 9:e94404. doi: 10.1371/journal.pone.0094404 24788351 PMC4006924

[67] Yan X Yang K Xiao Q Hou R Pan X Zhu X . Central role of microglia in sepsis-associated encephalopathy: from mechanism to therapy. Front Immunol. (2022) 13:929316. doi: 10.3389/fimmu.2022.929316 35958583 PMC9361477

[68] Lemstra AW Groen IWJ Hoozemans JJ van Haastert ES Rozemuller AJ Eikelenboom P . Microglia activation in sepsis: a case-control study. J Neuroinflamm. (2007) 4:4. doi: 10.1186/1742-2094-4-4 17224051 PMC1783646

[69] Kozlowski C Weimer RM . An automated method to quantify microglia morphology and application to monitor activation state longitudinally *in vivo*. PloS One. (2012) 7:e31814. doi: 10.1371/journal.pone.0031814 22457705 PMC3294422

[70] Guo JN Tian LY Liu WY Mu J Zhou D . Activation of the Akt/mTOR signaling pathway: a potential response to long-term neuronal loss in the hippocampus after sepsis. Neural Regener Res. (2017) 12:1832–42. doi: 10.4103/1673-5374.219044 29239329 PMC5745837

[71] Liu W Guo J Mu J Tian L Zhou D . Rapamycin protects sepsis-induced cognitive impairment in mouse hippocampus by enhancing autophagy. Cell Mol Neurobiol. (2017) 37:1195–205. doi: 10.1007/s10571-016-0449-x 27904994 PMC11482117

[72] O'Keefe GM Nguyen VT Benveniste EN . Class II transactivator and class II MHC gene expression in microglia: modulation by the cytokines TGF-beta, IL-4, IL-13 and IL-10. Eur J Immunol. (1999) 29:1275–85. doi: 10.1002/(SICI)1521-4141(199904)29:04<1275::AID-IMMU1275>3.0.CO;2-T 10229095

[73] Xu J Ling EA . Induction of major histocompatibility complex class II antigen on amoeboid microglial cells in early postnatal rats following intraperitoneal injections of lipopolysaccharide or interferon-gamma. Neurosci Lett. (1995) 189:97–100. doi: 10.1016/0304-3940(95)11462-6 7609927

[74] Li S Zhou Y Hu H Wang X Xu J Bai C . SIRT3 enhances the protective role of propofol in postoperative cognitive dysfunction via activating autophagy mediated by AMPK/mTOR pathway. Front Biosci (Landmark Ed). (2022) 27:303. doi: 10.31083/j.fbl2711303 36472103

[75] Wang R Hu X Liu S Wang J Xiong F Zhang X . Kaempferol-3-O-sophoroside (PCS-1) contributes to modulation of depressive-like behaviour in C57BL/6J mice by activating AMPK. Br J Pharmacol. (2024) 181:1182–202. doi: 10.1111/bph.16283 37949672

[76] Zhao P Li X Yang Q Lu Y Wang G Yang H . Malvidin alleviates mitochondrial dysfunction and ROS accumulation through activating AMPK-alpha/UCP2 axis, thereby resisting inflammation and apoptosis in SAE mice. Front Pharmacol. (2022) 13:1038802. doi: 10.3389/fphar.2022.1038802 36699054 PMC9868257

[77] Han Y Luo Y Sun J Ding Z Liu J Yan W . AMPK signaling in the dorsal hippocampus negatively regulates contextual fear memory formation. Neuropsychopharmacology. (2016) 41:1849–64. doi: 10.1038/npp.2015.355 26647974 PMC4869054

[78] Cajander S Tina E Backman A Magnuson A Stralin K Soderquist B . Quantitative real-time polymerase chain reaction measurement of HLA-DRA gene expression in whole blood is highly reproducible and shows changes that reflect dynamic shifts in monocyte surface HLA-DR expression during the course of sepsis. PloS One. (2016) 11:e154690. doi: 10.1371/journal.pone.0154690 27144640 PMC4856385

[79] Joshi I Carney WP Rock EP . Utility of monocyte HLA-DR and rationale for therapeutic GM-CSF in sepsis immunoparalysis. Front Immunol. (2023) 14:1130214. doi: 10.3389/fimmu.2023.1130214 36825018 PMC9942705

[80] Winkler MS Rissiek A Priefler M Schwedhelm E Robbe L Bauer A . Human leucocyte antigen (HLA-DR) gene expression is reduced in sepsis and correlates with impaired TNFalpha response: a diagnostic tool for immunosuppression? PloS One. (2017) 12:e182427. doi: 10.1371/journal.pone.0182427 28771573 PMC5542660

[81] Ting JP Trowsdale J . Genetic control of MHC class II expression. Cell. (2002) 109 Suppl:S21–33. doi: 10.1016/s0092-8674(02)00696-7 11983150

[82] Steimle V Otten LA Zufferey M Mach B . Complementation cloning of an MHC class II transactivator mutated in hereditary MHC class II deficiency (or bare lymphocyte syndrome). Cell. (1993) 75:135–46. doi: 10.1016/s0092-8674(05)80090-x 8402893

[83] Li G Mao Y Liao J Zhou Y . Integrated multiomics and Mendelian randomization identify CHIT1 as a novel sepsis biomarker and therapeutic target. Sci Rep. (2025) 15:15715. doi: 10.1038/s41598-025-99619-z 40325173 PMC12052846

[84] Cai L Rodgers E Schoenmann N Raju RP . Advances in rodent experimental models of sepsis. Int J Mol Sci. (2023) 24(11):9578. doi: 10.3390/ijms24119578 37298529 PMC10253762

[85] Giridharan VV Catumbela C Catalao C Lee J Ganesh BP Petronilho F . Sepsis exacerbates Alzheimer's disease pathophysiology, modulates the gut microbiome, increases neuroinflammation and amyloid burden. Mol Psychiatry. (2023) 28:4463–73. doi: 10.1038/s41380-023-02172-2 37452088 PMC10926876

[86] Corneo E Michels M Abatti M Vieira A Goncalves RC Gabriel FF . Enriched environment causes epigenetic changes in hippocampus and improves long-term cognitive function in sepsis. Sci Rep. (2022) 12:11529. doi: 10.1038/s41598-022-14660-6 35798809 PMC9262921

[87] Gonnert FA Recknagel P Seidel M Jbeily N Dahlke K Bockmeyer CL . Characteristics of clinical sepsis reflected in a reliable and reproducible rodent sepsis model. J Surg Res. (2011) 170:e123-34. doi: 10.1016/j.jss.2011.05.019 21737102

[88] Yeung RS Penninger JM Kundig T Khoo W Ohashi PS Kroemer G . Human CD4 and human major histocompatibility complex class II (DQ6) transgenic mice: supersensitivity to superantigen-induced septic shock. Eur J Immunol. (1996) 26:1074–82. doi: 10.1002/eji.1830260518 8647170

[89] Tuffs SW Dufresne K Rishi A Walton NR McCormick JK . Novel insights into the immune response to bacterial T cell superantigens. Nat Rev Immunol. (2024) 24:417–34. doi: 10.1038/s41577-023-00979-2 38225276

[90] Fukui Y . Functional expression of xenogenic mixed isotype molecule, DR alpha E beta, in transgenic mice with HLA-DRA gene on X chromosome. Fukuoka Igaku Zasshi. (1992) 83:80–90. 1534306

[91] Erickson MA Banks WA . Cytokine and chemokine responses in serum and brain after single and repeated injections of lipopolysaccharide: multiplex quantification with path analysis. Brain Behav Immun. (2011) 25:1637–48. doi: 10.1016/j.bbi.2011.06.006 21704698 PMC3389494

[92] Lin SC Hardie DG . AMPK: sensing glucose as well as cellular energy status. Cell Metab. (2018) 27:299–313. doi: 10.1016/j.cmet.2017.10.009 29153408

[93] Luo L Wu J Qiao L Lu G Li J Li D . Sestrin 2 attenuates sepsis-associated encephalopathy through the promotion of autophagy in hippocampal neurons. J Cell Mol Med. (2020) 24:6634–43. doi: 10.1111/jcmm.15313 32363721 PMC7299720

[94] Shu H Wang M Song M Sun Y Shen X Zhang J . Acute nicotine treatment alleviates LPS-induced impairment of fear memory reconsolidation through AMPK activation and CRTC1 upregulation in hippocampus. Int J Neuropsychopharmacol. (2020) 23:687–99. doi: 10.1093/ijnp/pyaa043 32516360 PMC7727489

[95] Chen T Chen X Zhang S Zhu J Tang B Wang A . The genome sequence archive family: toward explosive data growth and diverse data types. Genomics Proteomics Bioinf. (2021) 19:578–83. doi: 10.1016/j.gpb.2021.08.001 34400360 PMC9039563

[96] Database Resources of the National Genomics Data Center, China National Center for Bioinformation . Nucleic Acids Res. (2025) 53:D30–44. doi: 10.1093/nar/gkae978 39530327 PMC11701749

