# Supplementary figures and images for: HLA DP/DRA molecule regulates systemic inflammation and neuroinflammation, aggravates cognitive impairment and long-term anxiety in murine model of sepsis-associated encephalopathy
Source: Front Immunol. 2026 Jun 16;17:1798003. doi: 10.3389/fimmu.2026.1798003 (PMC13314453; doi:10.3389/fimmu.2026.1798003)

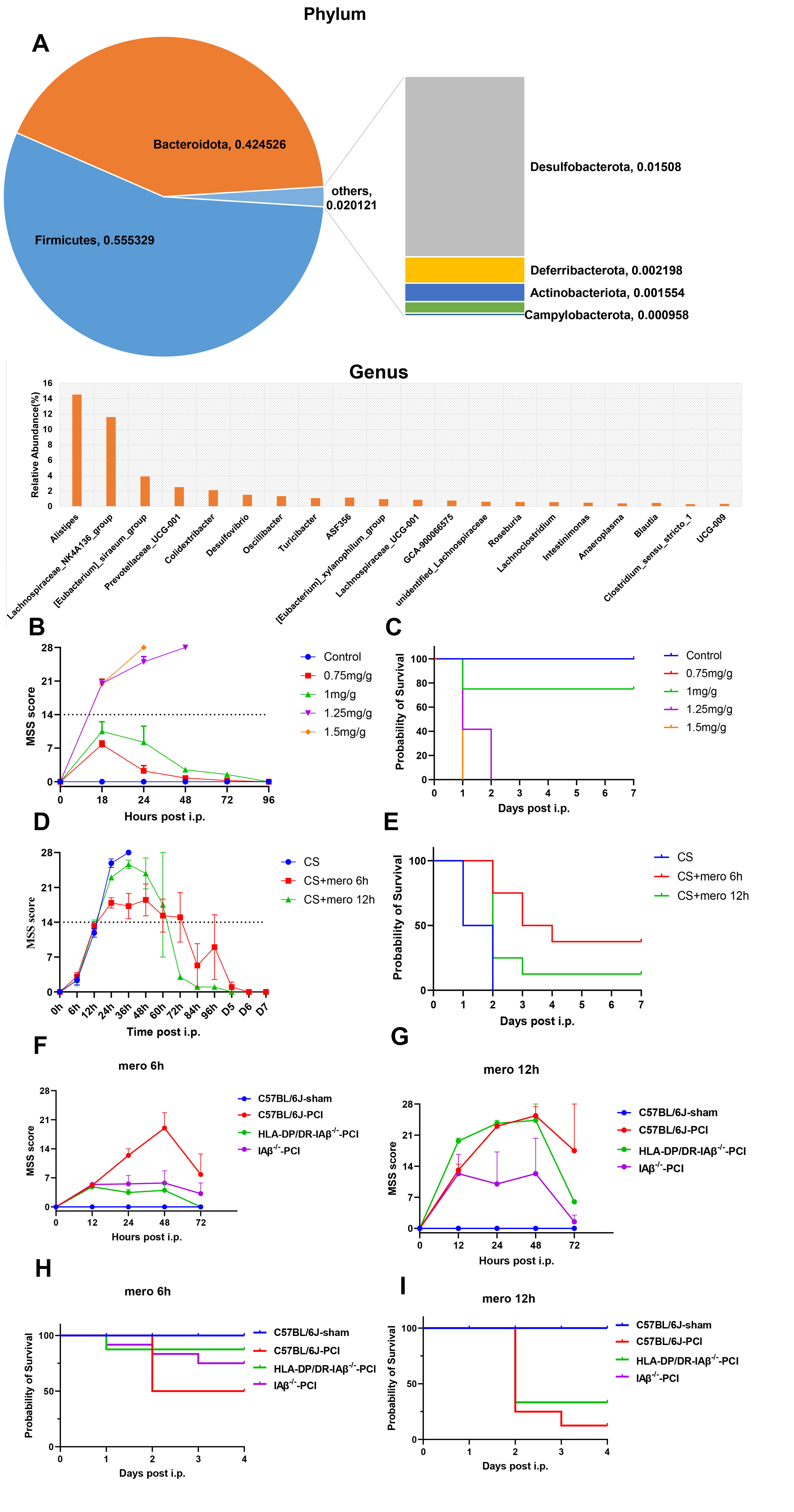

Supplement: Supplementary Figure 1 — (A) Relative abundances of bacteria in the cecal slurry used in this study at the phylum and genus levels. At the phylum level, Firmicutes and Bacteroidota collectively accounted for the majority of total abundance (98%). At the genus level, high relative abundances were observed for alistipes, lachnospiraceae_NK4A136_group, eubacterium_siraeum group, prevotellaceae_UCG-001, colidextribacter, desulfovibrio, oscillibacter and turicibacter. (B) Higher doses of CS slurry (CS; 1.25 and 1.5mg/kg) induced higher Murine Sepsis Score (MSS) (B). (C) Corresponding survival rate. (D, E) Animals received antibiotic treatment (meropenem, 20 mg/kg i.p.) starting at 6 and 12 h after 1.2 mg/kg CS injection; antibiotics were administered twice daily for 5 consecutive days (n = 8–10 per group). MSS (D) and survival rate (E) were monitored daily. (F–I) HLA-DP401/DRA-IAβ-/- and IAβ-/- mice received a lethal dose of CS (1.2 mg/kg) with antibiotic initiation at 6 h or 12 h post-injection. (F) mice with IAβ-/- background exhibited significantly lower MSS at the 6 h time point. (G) HLA-DP401/DRA-IAβ-/- and WT PCI mice showed higher MSS at the 12 h time point compared with IAβ-/- PCI mice. [file Image1.jpeg]

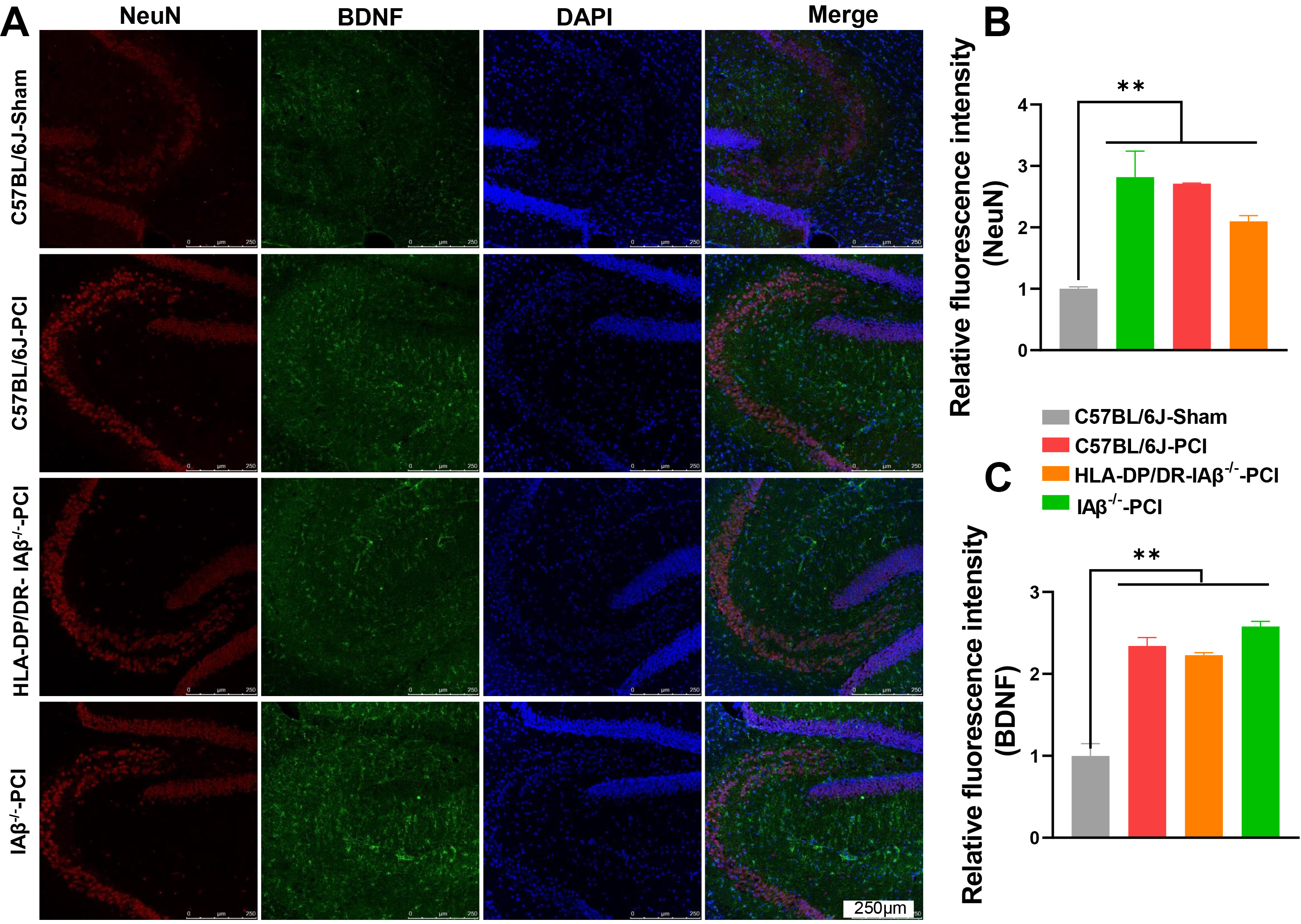

Supplement: Supplementary Figure 2 — Sepsis induce the rising expression of NeuN and BDNF in hippocampus from PCI mice. (A) Representative images of immunofluorescence staining of NeuN (red), BDNF (green), DAPI (blue) and colocalization in the hippocampus, scale bar = 50 μm. Quantification of the number of NeuN+ (B) and BDNF+ (C) cells in the hippocampus among the four groups. Data are presented as the mean ± SEM (n = 3 mice/group). **P < 0.01 vs. the indicated groups. [file Image2.jpeg]

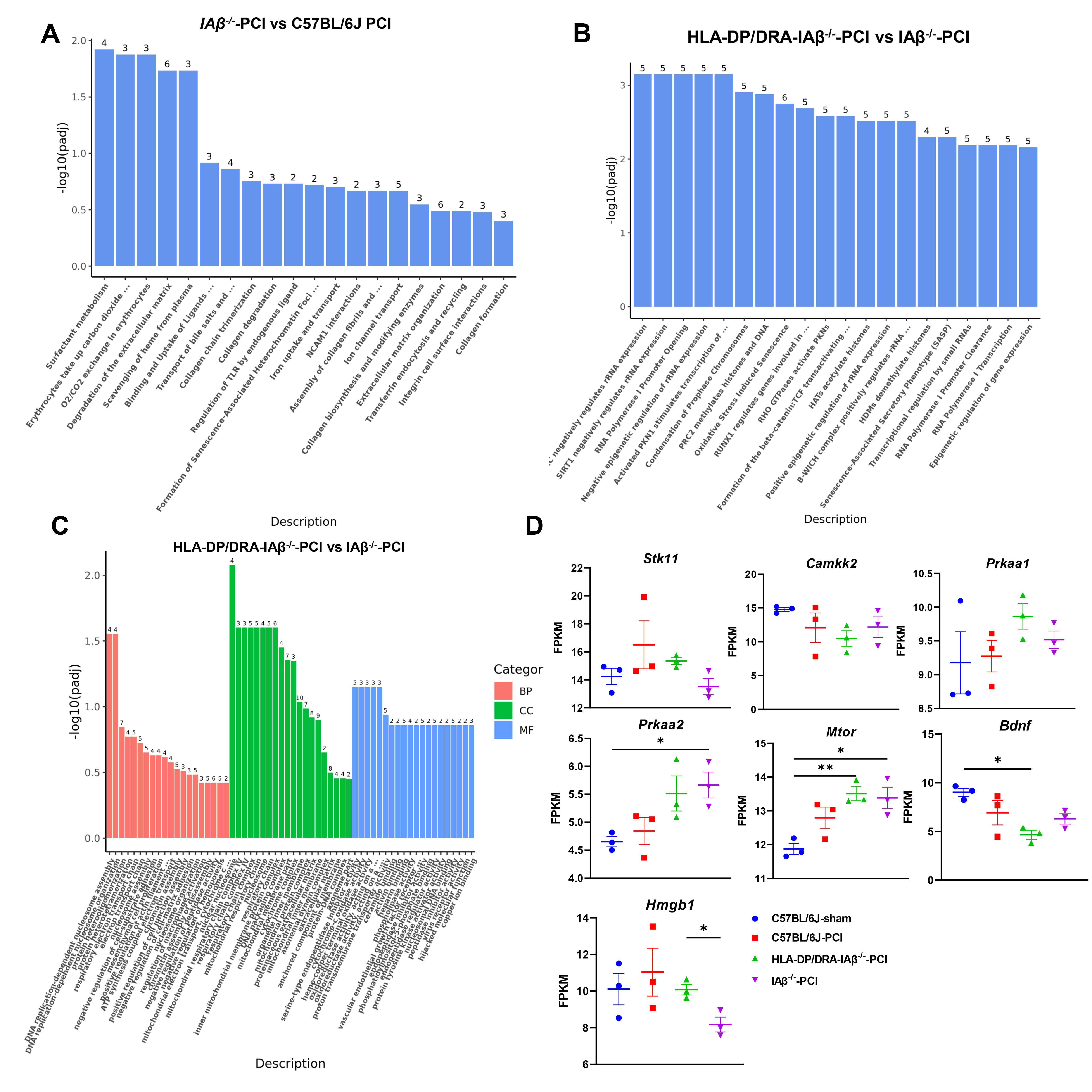

Supplement: Supplementary Figure 3 — Recktome and GO enrichment analysis. Rectome analysis for IAβ-/- PCI vs. C57BL/6J PCI mice (A), HLA-DP/DRA-IAβ-/- versus IAβ-/- PCI mice (B) in whole hippocampus tissue at day 3 following sepsis induction (n = 3, P < 0.05). (C) GO enrichment analysis for HLA-DP/DRA-IAβ-/- PCI mice versus IAβ-/- PCI mice in whole hippocampus tissue at day 3 following sepsis induction (n = 3, P < 0.05). (D) Expression of DEGs associated to mTOR, AMPK pathway after PCI- and sham-treated mice at day 3 following sepsis induction (n = 3, P < 0.05). [file Image3.jpeg]

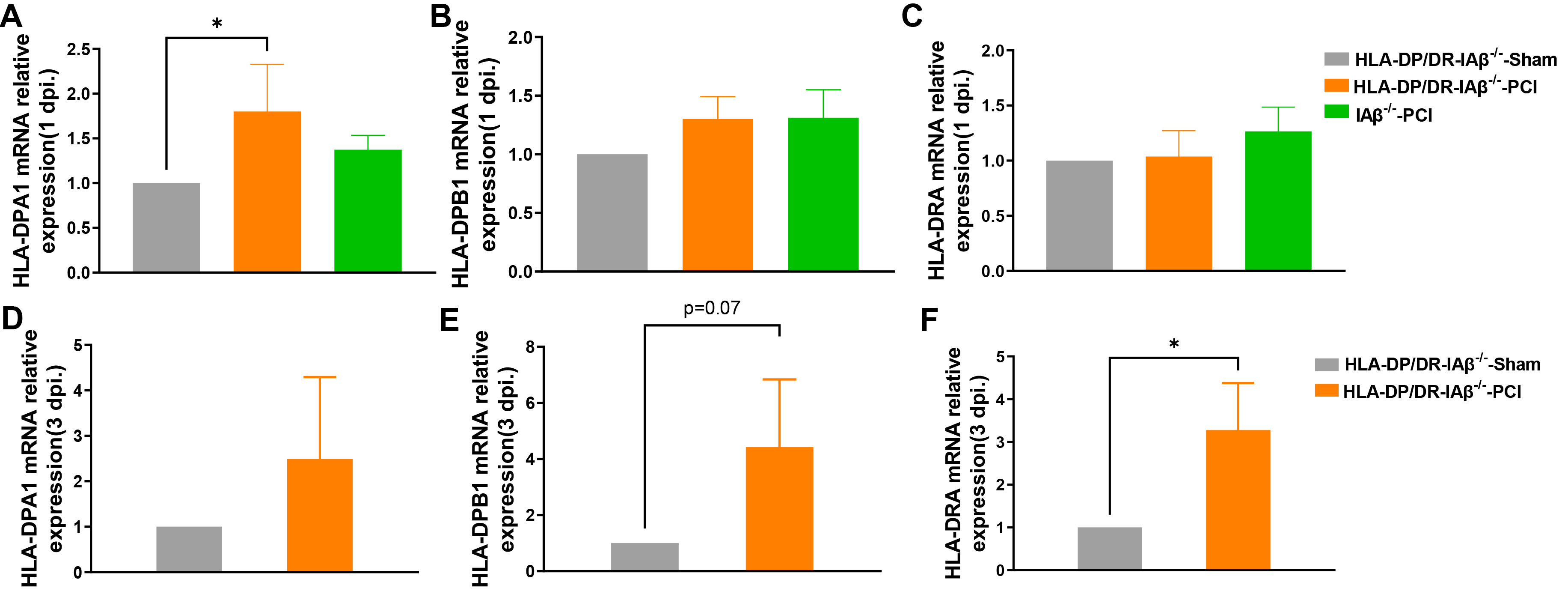

Supplement: Supplementary Figure 4 — Measurement of HLA-DP and -DRA mRNA levels in hippocampus tissues from HLA-DP/DRA-IAβ-/- PCI mice. The indicated tissue homogenates were used to measure the expression levels of the HLA-DPA1, -DPB1 and -DRA molecules by QPCR at D1 (A–C) and D3 (D–F) after CS injection. Note the increasing expression of HLA-DPB1 (E) and -DRA (F) in hippocampus at D3. Data was shown as the mean ± SEM of three individual mice. *P < 0.05. [file Image4.jpeg]

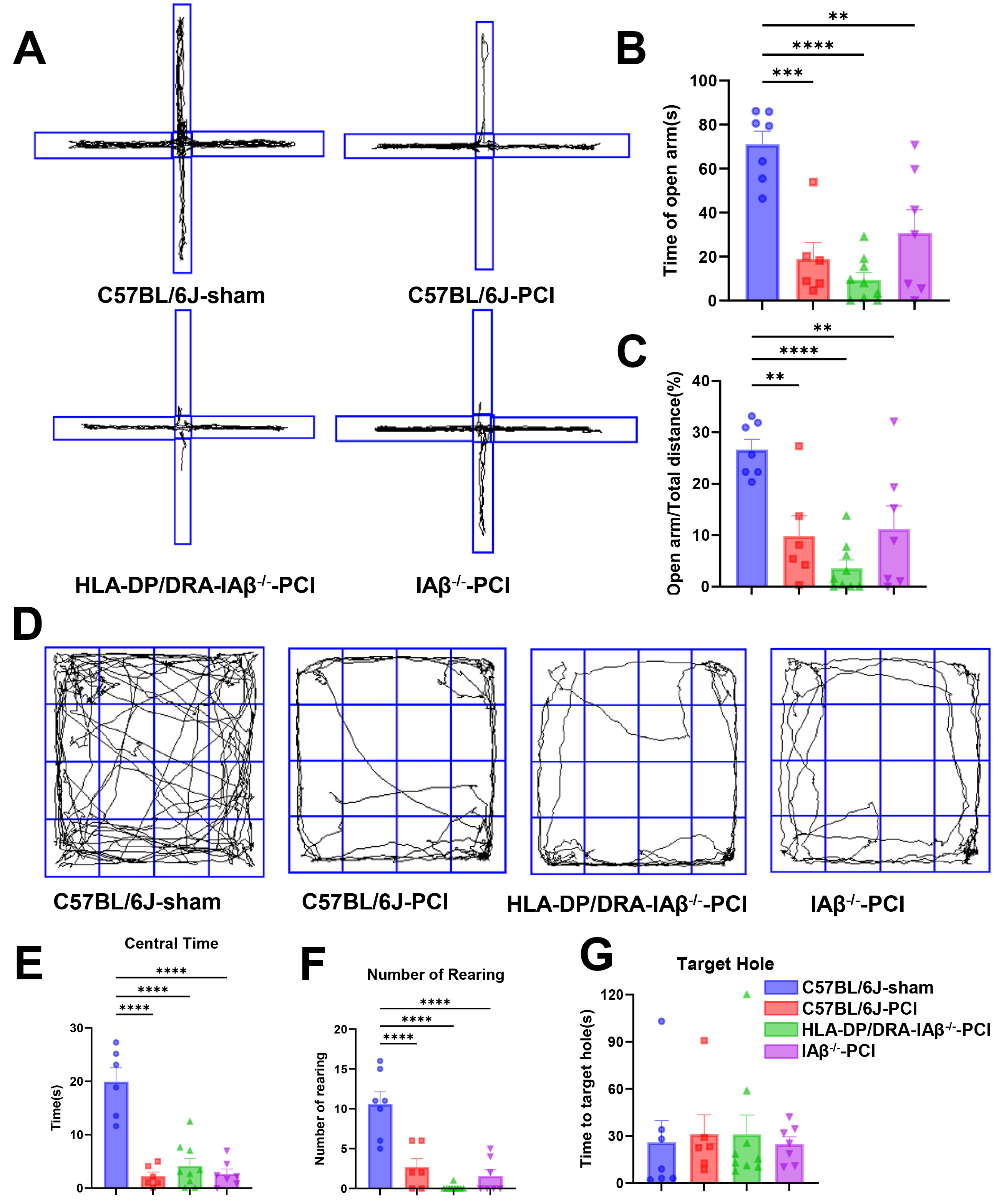

Supplement: Supplementary Figure 5 — Long-term anxiety persists in septic mice at D30 (A–C) In EPM, PCI mice spent less time and traveled fewer distance in open arms, compared with that in control mice, indicating that PCI mice had increased anxiety-like behavior. (D–F) In OF, PCI mice had lesser central time and had fewer rearing numbers. (G) In BM, PCI mice spent similar time to get the target hole with control mice, indicating a recovery of memory performance. Data are represented as mean ± SEM *P < 0.05, **P < 0.01, ***P < 0.001, ****P < 0.000. [file Image5.jpeg]

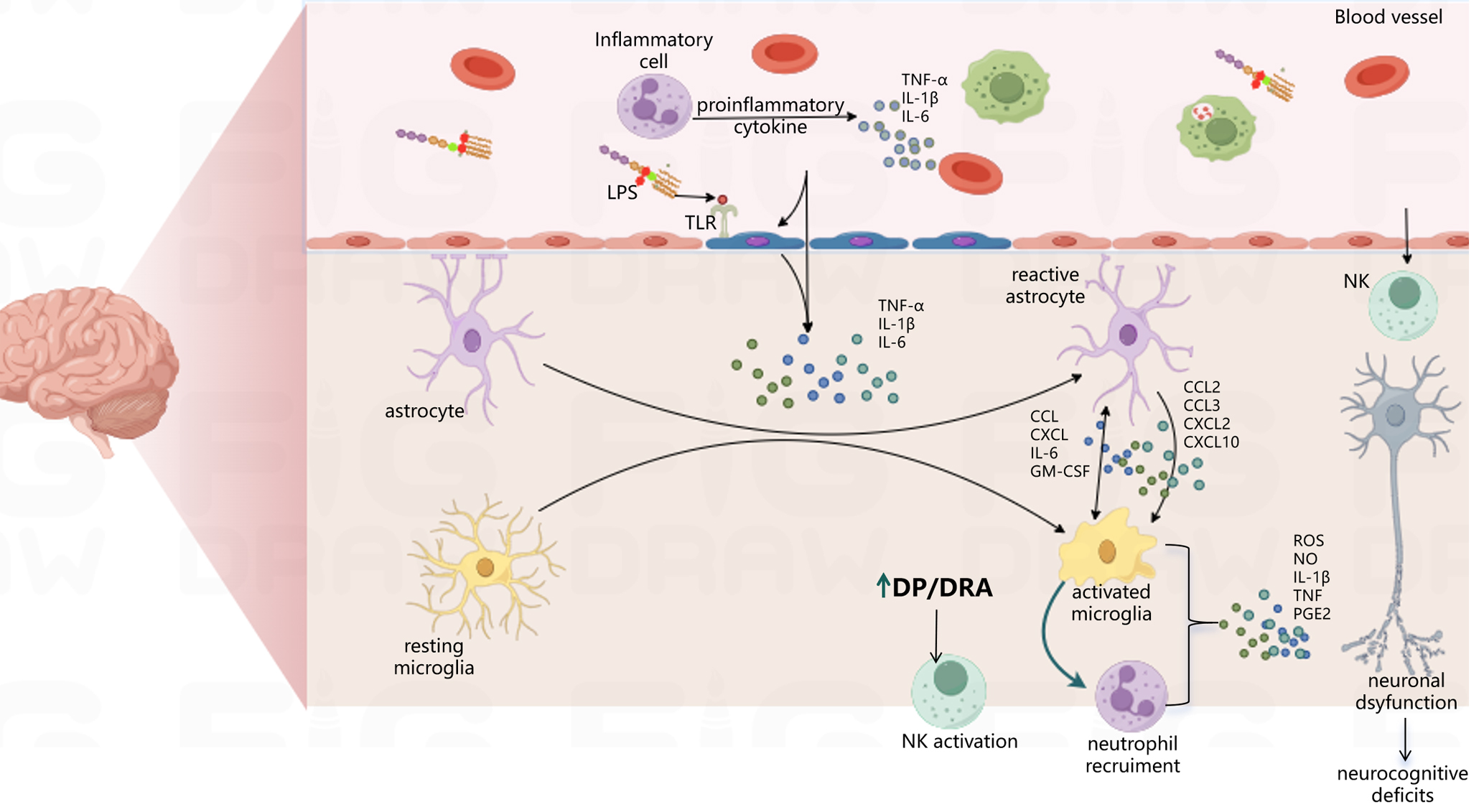

Supplement: Supplementary file 6 [file Image6.jpeg]
