# Supplementary material for: HLA DP/DRA molecule regulates systemic inflammation and neuroinflammation, aggravates cognitive impairment and long-term anxiety in murine model of sepsis-associated encephalopathy
Source: Front Immunol. 2026 Jun 16;17:1798003. doi: 10.3389/fimmu.2026.1798003 (PMC13314453; doi:10.3389/fimmu.2026.1798003)
Supplement: Supplementary file 11 [file Table4.docx]

Supplementary Table S1 PCR primers

| Gene | Sequence |  |
| --- | --- | --- |
| Mouse Tnf-α | Forward | AAGAGGCACTCCCCCAAAAGAT |
|  | Reverse | TCACCCCGAAGTTCAGTAGACA |
| Mouse IL-1β | Forward | CTTCAGGCAGGCAGTATC |
|  | Reverse | CAGCAGGTTATCATCATCATC |
| Mouse IL-6 | Forward | TACCACTCCCAACAGACC |
|  | Reverse | TTTCCACGATTTCCCAGA |
| Human Tnf-α | Forward | GAGCCAGCTCCCTCTATTTA |
|  | Reverse | GGGAACAGCCTATTGTTCAG |
| Human IL-1β | Forward | CATGGGATAACGAGGCTTATG |
|  | Reverse | CCACTTGTTGCTCCATATCC |
| Human IL-6 | Forward | CCTTCCAAAGATGGCTGAAA |
|  | Reverse | TGGCTTGTTCCTCACTACT |
| HLA-DPA1 | Forward | CAGACGCATAGACCAACAGG |
|  | Reverse | CAGGCTCCTTGGGAAACA |
| HLA-DPB1 | Forward | TACCCAGGCAGCATTCAA |
|  | Reverse | AAGGACAGACCCAGGTTTAGT |
| HLA-DRA | Forward | GTCATCTTCAGCATTTTCCA |
|  | Reverse | GTTTTATTCCAGAGGCATTG |
| CCL-2 | Forward | CTCTTCCTCCACCACCAT |
|  | Reverse | CTCTCCAGCCTACTCATTG |
| CCL-3 | Forward | CAGCGAGTACCAGTCCCTTT |
|  | Reverse | GCGCTGAGAAGACTTGGTTG |
| CXCL10 | Forward | TTGAGATCATTGCCACGAT |
|  | Reverse | CTCTGCTGTCCATCCATC |
| CXCL-2 | Forward | GCAAGGCTAACTGACCTGGAA |
|  | Reverse | CAACATCTGGGCAATGGAAT |
| G-CSF | Forward | CCTGGAGCAAGTGAGGAAGATC |
|  | Reverse | CGCTGGAAGGCAGAAGTGAA |
| CXCL-9 | Forward | GGCAGGTTTGATCTCCGTTC |
|  | Reverse | GGAGTTCGAGGAACCCTAGT |
| CXCL-1 | Forward | ACCCAAACCGAAGTCATAGC |
|  | Reverse | TGGGGACACCTTTTAGCATC |
| Lcn2 | Forward | ATATGCACAGGTATCCTCAG |
|  | Reverse | GAAACGTTCCTTCAGTTCAG |
| CCL-11 | Forward | CTGCTCACGGTCACTTCCTT |
|  | Reverse | TCTTTGCCCAACCTGGTCTT |
| Mouse Gapdh | Forward | TGGCCTTCCGTGTTCCTAC |
|  | Reverse | GAGTTGCTGTTGAAGTCGCA |
| Human Gapdh | Forward | GATTCCACCCATGGCAAATTC |
|  | Reverse | CTGGAAGATGGTGATGGGATT |

Supplementary Table S2 Primary antibodies

| Name | Manufacturer | Catalog | Application | dilution rate |
| --- | --- | --- | --- | --- |
| nNOS | Servicebio | GB11145 | WB | 1：500 |
| eNOS | Servicebio | GB11086 | WB | 1：500 |
| AMPK | Servicebio | GB112669 | WB | 1：500 |
| p-AMPK | Servicebio | GB114323 | WB | 1：800 |
| mTOR | Servicebio | GB111839 | WB | 1：500 |
| p-mTOR | Servicebio | GB114489 | WB | 1：500 |
| Caspase-3 | CST | #9661 | WB | 1：500 |
| β-Actin | TransGen Biotech | HC201-01 | WB | 1：500 |
| Iba1 | Abcam | ab178846 | IF | 1:1000 |
| NeuN | MILLPORE | LV1634819 | IF | 1:200 |
| BDNF | BOSTER | BM2020 | IF | 1:100 |
